# Sliding perspectives: dissociating ownership from self-location during full body illusions in virtual reality

**DOI:** 10.3389/fnhum.2014.00693

**Published:** 2014-09-11

**Authors:** Antonella Maselli, Mel Slater

**Affiliations:** ^1^EVENT Lab, Facultat de Psicologia, Universitat de BarcelonaBarcelona, Spain; ^2^Institució Catalana Recerca i Estudis AvancatsBarcelona, Spain

**Keywords:** ownership, self-location, full body ownership illusions, out of body experience, multisensory integration, visual perspective, cross-modal congruency effect, virtual reality

## Abstract

Bodily illusions have been used to study bodily self-consciousness and disentangle its various components, among other the sense of ownership and self-location. Congruent multimodal correlations between the real body and a fake humanoid body can in fact trigger the illusion that the fake body is one's own and/or disrupt the unity between the perceived self-location and the position of the physical body. However, the extent to which changes in self-location entail changes in ownership is still matter of debate. Here we address this problem with the support of immersive virtual reality. Congruent visuotactile stimulation was delivered on healthy participants to trigger full body illusions from different visual perspectives, each resulting in a different degree of overlap between real and virtual body. Changes in ownership and self-location were measured with novel self-posture assessment tasks and with an adapted version of the cross-modal congruency task. We found that, despite their strong coupling, self-location and ownership can be selectively altered: self-location was affected when having a third person perspective over the virtual body, while ownership toward the virtual body was experienced only in the conditions with total or partial overlap. Thus, when the virtual body is seen in the far extra-personal space, changes in self-location were not coupled with changes in ownership. If a partial spatial overlap is present, ownership was instead typically experienced with a boosted change in the perceived self-location. We discussed results in the context of the current knowledge of the multisensory integration mechanisms contributing to self-body perception. We argue that changes in the perceived self-location are associated to the dynamical representation of peripersonal space encoded by visuotactile neurons. On the other hand, our results speak in favor of visuo-proprioceptive neuronal populations being a driving trigger in full body ownership illusions.

## Introduction

A large body of experimental research in cognitive neuroscience has exploited bodily illusions to study bodily self-consciousness. This work has established a tight link between self-consciousness and the processing of multisensory bodily signals performed in specific brain areas (Makin et al., 2008; Ehrsson, 2011; Blanke, 2012). It further allowed disentangling distinct components of bodily self-consciousness and their candidate neural representations (Longo et al., 2008; Blanke and Metzinger, 2009; Tsakiris et al., 2010).

The present study focuses on the mutual relation between two important components of bodily self-consciousness: the feeling of owning a body (body *ownership*) and the experience of the body occupying a given portion of space in the environment (*self-location*). Although in normal conditions *ownership* and *self-location* are tightly tied, recent experimental work has shown that these two components are, at least partially, separable (Longo et al., 2008). However, so far it has not been explored whether these two components are intrinsically coupled or are rather dissociable despite their strong coupling (Serino et al., 2013). In this article we present a study performed with the support of virtual reality (VR) technology, and designed to specifically test the hypothesis that, under specific experimental conditions, *ownership* and *self-location* can be selectively altered. The rationale behind the current study is based on a series of previous studies that are reviewed in the following.

### Experimental manipulations of the sense of self-location

*Self-location* has been mainly investigated through experimentally induced out-of-body experiences (OBE). The experimental paradigm to induce an OBE in healthy subject consists in participants wearing a head-mounted display (HMD) occluding the physical body and displaying the back of their own body -or that of a mannequin- as filmed from a distance of roughly two meters. The visual perspective then coincides with the location of the real body and is dissociated from the location of the virtual body seen through the HMD. The illusion is then triggered through congruent visuotactile stimulation on the real and fake body. When synchronous visuotactile stimulation is delivered on the back (Lenggenhager et al., 2007), most participants experience the illusion of looking at their body from the outside, similarly to what is reported in OBEs of neurological origin (Blanke and Mohr, 2005). This illusion is associated with illusory changes in *self-location*, with a systematic shift from the participant's visual perspective toward the seen body. Such a drift has been consistently found throughout a variety of assessments: the “walking task ” (Lenggenhager et al., 2007) and the “mental ball dropping” task (Lenggenhager et al., 2009) provided explicit measures of the perceived *self-location* in external and body-centered reference frames respectively, while the crossmodal congruency task (CCT) was used to implicitly assess *self-location* through the localization of tactile events in the external space (Aspell et al., 2009).

Functional magnetic resonance imaging (fMRI) revealed that these illusory changes in *self-location* correlate with activity in the temporo-parietal junction (TPJ) and the extrastriate body area (EBA) (Ionta et al., 2011). The implication of the TPJ was consistently found in lesion studies on neurological patients suffering aberrations in *self-location* (Blanke et al., 2004). Focal electrical stimulation of the TPJ was further shown to systematically trigger OBE (Blanke et al., 2002).

In a similar experimental setting, OBE illusions have been alternatively triggered by delivering synchronous visuotactile stimulation on the chest while displaying the visual stimuli at the location of the occluded physical body (Ehrsson, 2007). In contrast to what found in the “back stroking” case, participants tended to feel located at the position of the physical body; this was implicitly assessed by asking participants to rate how much they felt at the location were they saw their body or at the camera's (i.e., the physical body's) location (Guterstam and Ehrsson, 2012). The difference is probably due to the fact that, in the “chest stroking” case there is no spatial conflict between visual and tactile cues. In the “back stroking” case, it probably the spatial misalignment of visual and tactile synchronous cues that induces a recalibration in the perceived *self-location*.

### Experimental manipulations of the sense of ownership

The sense of ownership toward a full body has been largely studied making use of the full body ownership illusion (FBOI), defined as the continuous and consciously impermeable feeling that a virtual/fake full body, including all its body parts, belongs to us in its integrity.

Built up on the rich experimental literature on the rubber hand illusion (Botvinick and Cohen, 1998), the experimental paradigm to elicit a FBOI consists in outfitting participants with an immersive HMD that occludes the real body and displays in its place a fake humanoid body, which may be a filmed mannequin (Petkova and Ehrsson, 2008) or a virtual character (Slater et al., 2010). Unlike the case of OBE, participants have thus a first person perspective (1PP) of the fake body usually displayed in the same posture as the real body. This setup provides participants with congruent visuo-proprioceptive cues, i.e., visual information about the virtual body's spatial configuration are congruent with perceptual cues about the body posture delivered by muscle spindle and join receptors. In the case of a static filmed mannequin, synchronous visuotactile stimulation was found to be the necessary trigger for eliciting the illusion (Petkova and Ehrsson, 2008). However, it was shown that other modalities of multisensory/multimodal correlations could trigger the illusion with no need for synchronous visuotactile stimulation. In particular, visuomotor correlations are extremely efficient in eliciting a FBOI (Slater et al., 2010; Kokkinara and Slater, 2014), thanks to the rich information processing involved in the sensorimotor control loop. On the other hand, if the fake body has a highly realistic humanoid appearance, the illusion may be elicited by congruent visuo-proprioceptive cues alone, with no need for further multimodal correlations (Maselli and Slater, 2013).

Healthy subjects experiencing a FBOI report that the seen fake body was perceived as if it was their own body and as if perceptual experiences, e.g., tactile events, were generated from the fake body. Apart from self-reports and questionnaires, the FBOI is indeed evaluated by monitoring autonomic responses to events threatening the fake body. For example, when attacking or threatening the integrity of the fake body during a FBOI, significant increases in skin conductance response (Petkova and Ehrsson, 2008) and in heart rate deceleration (Slater et al., 2010; Maselli and Slater, 2013) have been found. Importantly, the measured change in autonomic responses is positively correlated with the intensity of the illusion inferred from questionnaire scores. By monitoring brain activity with fMRI during a FBOI, it was found that the illusion correlates with activity in bilateral ventral premotor cortex (vPMc), left intraparietal cortex and left putamen (Petkova et al., 2011a). Interestingly, the ventral premotor cortex activation was found to be particularly associated with the construction of the unitary experience of a full body, ensuing from the merging of the sense of ownership of different body parts. No implication of the TPJ during a FBOI has been reported.

An alternative approach to explore the sense of ownership, complementary to the FBOI, is the “chest stroking” OBE illusion described in the previous section (Ehrsson, 2007; Guterstam and Ehrsson, 2012). Autonomic responses to threat events have shown how, in this illusion, the sense of ownership is “attached” to the not visible real body located at the position of the visual perspective (Ehrsson, 2007; Guterstam and Ehrsson, 2012), while the sense of ownership toward the body seen in the extra-personal space is disrupted (Guterstam and Ehrsson, 2012).

### Do ownership and self-location have different neural representations? hints from full body illusions

When comparing results from the OBE and FBOI experiments discussed above, several hints emerge suggesting that *ownership* and *self-location* might have different origins and neural representations (Maselli and Slater, 2013; Serino et al., 2013).

First, as discussed above, fMRI studies revealed that OBE and FBOI are associated with the activation of different brain areas (Ionta et al., 2011; Petkova et al., 2011a). Second, the experimental paradigms used for inducing OBE and FBOI were developed to explicitly assess either changes in *self-location* or in *ownership*, and not both at the same time.

In 1PP FBOI, self-location is not altered at all as the real and virtual body are collocated. On the other hand, the OBE experiments reviewed above have been mainly designed to accurately measure changes in the perceived *self-location*. The concurrent feeling of *ownership* toward the body seen in the extra-personal space was mainly assessed through questionnaire and free reports. When pooled together, data from questionnaires and self-reports in “back stroking” OBE show that illusory changes in *self-location* are not systematically associated with the sense of *ownership*: self-reports from some participants (Ionta et al., 2011) suggest that this illusion is associated with self-recognition rather than with the experience of owning the virtual body (e.g., “*I had the impression of being touched by the stick as if I was between two mirrors and I could see my back*”; “*I had the impression of watching a photo of myself…*”). So it is possible that questionnaire data also reveal an enhanced sense of self-recognition rather than ownership toward the virtual body (Petkova et al., 2011b; Maselli and Slater, 2013). This possibility is further supported by the fact that participants score higher to the question “It felt as if the virtual body/mannequin was my body,” when they see their own body rather than a mannequin filmed from the back (Lenggenhager et al., 2007). This modulation with the appearance of the virtual body is not found instead in 1PP illusion, in which the virtual body could importantly differ from the real in terms of age, gender and race, without reducing the illusion (e.g., Slater et al., 2010; Peck et al., 2013).

Only with few studies attempted so far to assess *ownership* in the “back stroking” OBE paradigm through autonomic measures: widespread decrements in body temperature associated with this OBE illusion have been recently reported (Salomon et al., 2013) and, based on previous RHI analogous results (Moseley et al., 2008), interpreted as a shift of the sense of ownership from the real to the virtual body. However, these results are not conclusive, as the effects found were smaller than the thermometer's resolution. Pomés and Slater (2013) measures instead heart rate deceleration (HRD) and movements in responses to a threat to the virtual body. Although no significant modulation of the HRD was found, their multivariate analysis suggests that the sense of ownership can positively contribute to sensations of drifting toward the virtual body. Still, these results do not provide any direct evidence for modulations of *ownership* being systematically driven by an altered sense of *self-location*.

Upon direct comparison between 3PP and 1PP conditions, it has been systematically found that *ownership* is significantly suppressed in 3PP with respect to 1PP (Slater et al., 2010; Petkova et al., 2011b; Maselli and Slater, 2013). Results from the “chest stroking” OBE illusions further support these finding, showing how, in this full body illusion the sense of ownership toward the real body seen from a 3PP is disrupted (Guterstam and Ehrsson, 2012).

In summary, no direct experimental evidence is currently available for effective changes in *self-location* driven by OBE illusions to imply systematic changes in *ownership*. This scenario yields the motivation for the present study. In fact, the current experimental evidence suggests that *ownership* and *self-location* may have different neural representations and that they may be -at least to some extent- dissociated from each other. In this study we explicitly tested this hypothesis and further addressed the issue of how *ownership* and *self-location* interact when both are simultaneously altered in controlled experimental setups. The study is based on a set of three independent experiments conducted with the support of immersive virtual reality.

## The cross-modal congruency task as a measure of self-location

An important part of the results presented in this work relies on the use of the cross-modal congruency task (CCT). In this section we give a short introduction to clarify the principles that validate the CCT, under specific configurations, as a tool to measure *self-location* in external space.

The CCT is a standard psychophysics test that has been designed to study interactions between vision and touch (Spence et al., 2008). The task consists of discriminating the elevation of perceived target vibrations in the presence of visual distracter stimuli (Driver and Spence, 1998b). In the classical configuration an array of four vibrators and four light emitting diodes are arranged on the participant's hands (two on the indexes and two on the thumbs). On each trial a vibration and a light flash are presented and the participant has to make a rapid judgment on whether the vibration was up (on the index) or down (on the thumbs), irrespectively of the side and of the location where the flashing light switched on. A large number of studies have consistently shown that vibrotactile discrimination is delayed and less accurate when the distracting visual cue is incongruent in elevation with the target vibration. The effect is quantified in terms of the cross-modal congruency effect (CCE), defined as the difference in performances (e.g., response times or error rates) between incongruent and congruent trials. Because visuotactile interactions are stronger when the visual cue is presented closer to the target tactile stimulation, i.e., on the same side/hand, CCE values are typically larger when computed for trials with visual and tactile cues on the same side with respect to the opposite side (Spence et al., 2004).

Three different processes have been proposed as responsible for the observed visuotactile interaction: cuing of exogenous spatial attention, multisensory integration (MSI), and response competition (light distracters priming the response) (Spence et al., 2004). These different mechanisms may dominate the CCE or contribute to it in combination with others, depending on the time separation, i.e., the stimulus onset asynchrony (SOA), between the target and the distracter stimulus. MSI mechanisms dominate the CCE for SOA smaller than 100 ms (irrespectively of the stimuli order), while for SOAs outside this range the effect is mainly driven by exogenous attention or priming effects (Shore et al., 2006).

Using an appropriate SOA, the CCT can be therefore used as a tool for detecting the MSI of visual and tactile stimuli. The crossmodal effect found in a variety of spatial configurations can be indeed explained on the basis of the known spatial properties of bimodal visuotactile (VT) neurons found in the human and non-human primate's brain: the effect is preserved when the hands are crossed (Driver and Spence, 1998a; Maravita et al., 2003), showing how visual distracters are processed in the corresponding hand-centered reference frame, rather than in retinotopic coordinates. An analogous modulation is found in VT neural populations having visual receptive fields (vRFs) that shift in space along with body movements (Graziano and Gross, 1993; Duhamel et al., 1998; Lloyd et al., 2003; Brozzoli et al., 2012). Interestingly, crossmodal interactions were found also when the visual distracters were located on tools that have been actively manipulated (Maravita et al., 2002). This provided an additional behavioral counterpart for the properties of parietal VT neurons in the macaque monkey whose vRFs extend outwards after active tool manipulation (Iriki et al., 1996).

All these results are crucial in pointing out how the CCT can be exploited to infer the location of tactile events as perceived in external space. Following this rationale, previous studies used the CCT to test the spatial recalibration of the limb position occurring during a rubber hand illusion (RHI): effective crossmodal interactions were found despite the fact that vibrators and visual distracters (located on the rubber hands) were not aligned in external space (Pavani et al., 2000; Zopf et al., 2011), but only when the illusion was experienced. These results showed a recalibration of the perceived location the tactile stimuli, hence of real hands, toward the location of the rubber hands.

Similarly, the CCT has been adopted to infer shifts in the perceived location of the whole body, i.e., to test *self-location*, during OBEs (Aspell et al., 2009), with the arrays of lights and vibrators located on the back of the virtual and physical bodies respectively. An effective crossmodal interaction was found during the experienced OBE illusion, but only adopting a SOA of 233 ms, which falls in the regime in which crossmodal congruency effects are dominated by exogenous spatial attention rather than by MSI (Shore et al., 2006). No effect was found however for SOA range dominated by MSI effects, thus the interpretation of Aspell et al.'s results as due to illusory change in *self-location* is not straightforward.

## Materials and methods

### Participants

A total of 51 subjects were recruited for the study. Experiment 1 involved 15 naïve participants (12 females) with average age 21.6 years (±3.7 years SD); Experiment 2, 17 naïve participants (8) with average age 20.4 years (±2.3 years SD); Experiment 3, 19 naïve participants (10 females) with average age 22.7 years (±5.0 years SD). Participants in all the three groups had little or no previous experience in virtual reality (scores to the question “Have you ever experienced virtual reality before?” on a Likert scale from 1 to 7 had a median of 1 and interquartile range (IQR) of 1 for all the three experiments). All participants had normal or corrected to normal vision and had no history of neurological or psychiatric disorders. Participants signed an informed consent form before taking part to the experiment, and received a compensation of €10 after completion. The experimental protocol was approved by the “Comité Ético de Investigación” of the University of Barcelona, in line with the institutional ethics and national standards for the protection of human participants. In accordance with ethical commitments, all participants were contacted by email within 3–6 weeks after the experiment, and were asked whether they had any positive or negative thoughts about their experience in the experiment. None of the participants reported negative post-experimental sensations.

### Materials

**Figure 1:**
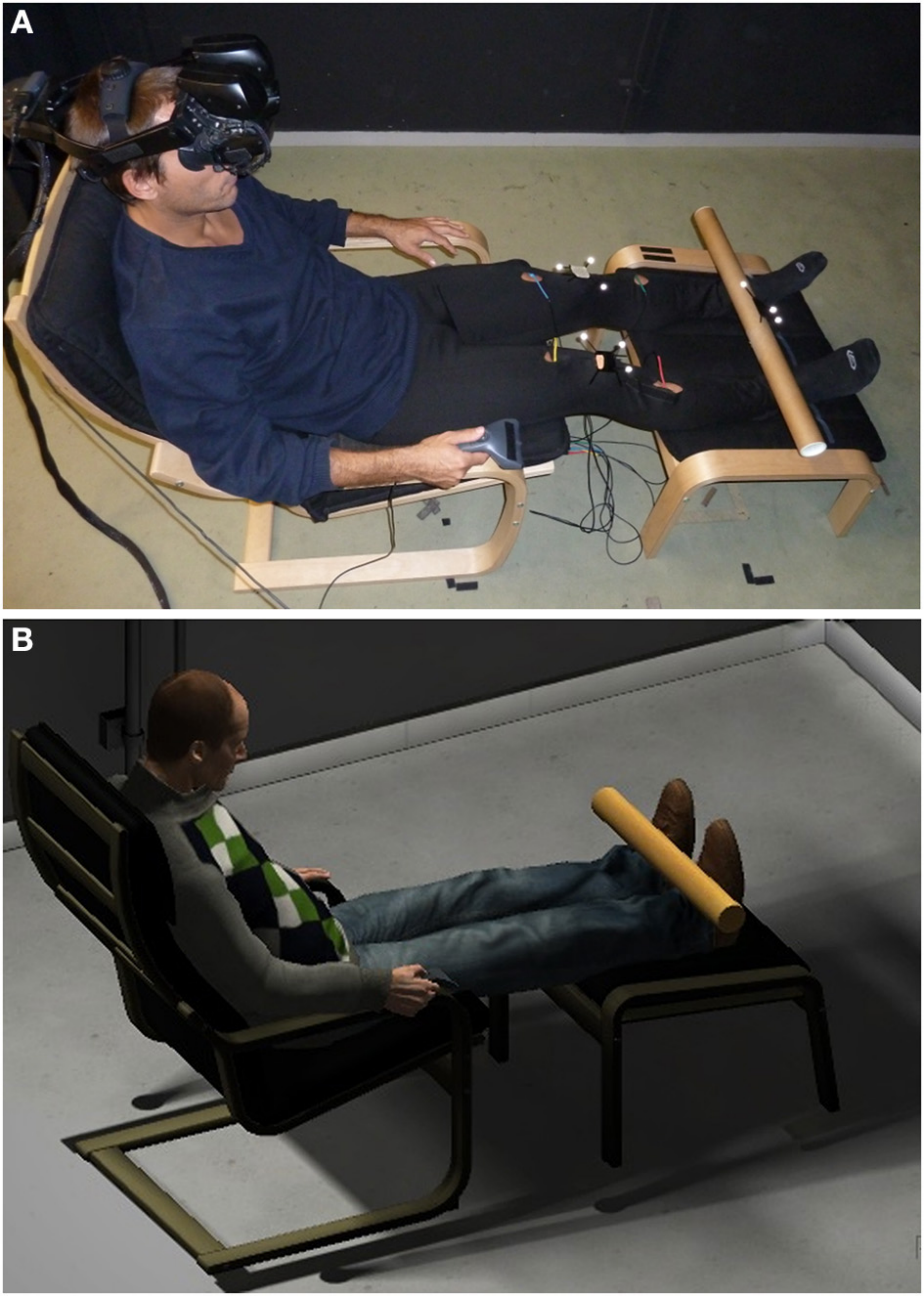
**Experimental setup**. The virtual environment **(B)** was a replication of the laboratory in which the experiment took place **(A)**. Participants experienced the environment through a stereo wide field-of-view head mounted display. Four vibrators, controlled by an Arduino board, were fixed on the participant legs for the cross-modal congruency task. A cardboard tube was used to deliver continuous visuotactile stimulations symmetrically on the two legs in some of the experimental conditions.

Experiments were implemented and conducted with the support of VR technology. The virtual environment was a faithful reproduction of the laboratory in which the experiments took place, modeled in 3D Studio Max by a graphics artist (Figure 1). The environment was controlled with the Unity^1^ platform. Participants entered the virtual environment through a stereo NVIS nVisor SX111^2^ HMD (dual SXGA displays with 76°H × 64°V field of view (FOV) per eye, with 50°(66%) of overlap in the horizontal axis, resulting in a total field of view of 102° horizontal and 64° vertical; image resolution: 1280 × 1024 per eye; frame rate: 60 Hz). Head tracking was performed by a six degree of freedom (DoF) Intersense IS-900 device based on a coupling of infrared monitoring and inertial technology^3^. The tracked data were streamed in real time using the Virtual Reality Peripheral Network^4^ (VRPN) protocol to the Unity project.

Visuotactile stimulation was delivered on the participant legs with a tracked cardboard tube, 70 cm in length and 5 cm in diameter. The tube's movements were tracked using the Tracking Tools toolkit of Optitrack^5^, a system form object motion tracking that uses 12 infrared cameras. Data from a 3D optical marker attached to the physical tube were streamed in real time (via the VRPN) to the Unity project and were used to control the movements of a virtual tube. Two optical markers were used to register the exact position of the knees of the participants with those of the virtual avatar, and other two were used in Experiment 3 for monitoring the position of the two hands. The length of the avatar's legs was scaled so to match the actual legs length for each participant. This allowed a precise colocation of the physical and virtual bodies in the 1PP condition, an exact spatial overlap of the vibrators arranged on the real legs and the virtual light distracters (LD), and further an accurate displacement of the virtual tube, which assured it to be displayed at exactly the same location on the real and virtual legs.

A set of four small vibrating motors^6^ was used for the CCT. The motors (3 V, 100 mA, 50 Ω, 1200 ± 300 r.p.m.) were controlled through an Arduino board connected to the Unity project. Participants used an Intersense joystick to give responses in the CCT and in the “Tube Task” described below.

### Response variables: measures, data processing and statistical methods

#### Crossmodal congruency test (CCT)

The CCT was used in our experiments to test possible shifts in the perceived *self-location* toward the virtual body. The rational for this choice has been exposed in details in Section The Cross-Modal Congruency Task as a Measure of Self-location. The array of LDs/vibrators used for the CCT was arranged on the virtual/physical legs. This novel configuration had been set to detect effects of multisensory integration of tactile events on the real body and visual events on the virtual body, while having different visual perspectives of the virtual body. The four vibrators were positioned on the participant's legs using elastic bands: two on the thighs, 10 cm above the knees, and two on the external side of the shins (on the tibialis anterior muscle), 10 cm below the keens. Four virtual LDs (round red lights with ~120 mm^2^ surface) were located in the corresponding positions on the virtual body. Each trial of the CCT consisted of a visual cue switching on during 33 ms and followed, after a 50 ms SOA, by a target vibration of 100 ms. Due to different delays in the video stream to the HMD and in the vibrators' activation (measured in the laboratory with an oscilloscope device), the effective SOA was about 20 ms with the light stimuli leading vibrations. Participants were instructed to keep their gaze on a fixation point (a blue dot in the middle of the LDs array) and, for each trial, to indicate as fast and as more accurately as possible whether the delivered target vibration was above (i.e., on the thighs) or below (i.e., on the lower legs), irrespectively of the side and of the LD's location. Participants gave responses using the upper (for above) and lower (for below) buttons of a joystick held in their right hand.

Each CCT run consisted of 180 trials: 160 experimental trials, 40 for each of the four possible combinations of LD and vibration locations, plus 20 “no-go” trials. In the “no-go” trials the fixation point changed color (from blue to fuchsia) and participants were instructed to stop giving responses even if target vibrations were released. “No-go” trials were included in order to make sure that participants kept their eyes open and focused on the fixation point throughout the whole duration of the task (Zopf et al., 2010); subjects that responded to more than 30% of the no-go trials were excluded from the CCT data analysis. The 180 trials were presented in a random order.

The CCT data were initially processed to extract the average response time (RT) and error percentage (EP) for each participant as a function of *side* (left leg vs. right leg), *congruency* (in elevation) and experimental *condition*. Trials with incorrect responses or with RT larger than 1500 ms or smaller than 200 ms, were discarded. The two measures were then combined into the inverse efficiency (IE) score, defined as the ratio between the reaction time and the percentage of correct responses [IE = RT/(1 − EP)], which provides the most informative summary of the data available and compensate for possible speed-accuracy trade-offs (Townsend and Ashby, 1983; Kennett et al., 2001; Spence et al., 2001).

We chose to perform the statistical analysis on the IE data because of the better informative content of this variable with respect to the more commonly used RT (Bruyer and Brysbaert, 2011).

The IE data were first analyzed in a Three-Way repeated measures ANOVA (3 × 2 × 2) with interactions, with factors *condition, side* and *congruency*. This can detect the cross-modal interactions in terms of a main effect of congruency -because of the longer response times and the higher error rate in incongruent trials- and any interaction between *congruency* and *side* -which shows that the cross-modal interaction is stronger for stimuli close in space-. A significant interaction among the three factors would detect critical differences among the various experimental. Pairwise comparisons were then performed running a One-Way repeated measurement ANOVA on CCE-IE (the CCE calculated on the performance variable IE) with the single factor *side*: as discussed above, a significant modulation of the CCE by *side* gives hints for a perceived spatial alignment between the arrays of vibrators and LDs, and in turns provides a proxy for the perceived *self-location*.

#### The “Tube Task” (TT)

The “Tube Task” (TT) is a novel test designed to gather measures of *ownership* and *self-location*. Participants were instructed to use a joystick to adjust the size (Experiment 1) the size plus the later position (Experiment 2) of a virtual tube, so to match with its extremities the perceive locations of their external ankles.

During the task no virtual body was displayed and a blue virtual tube was the unique visible object in the environment. The tube was displayed horizontally so to pass through the participant's ankles locations. At the beginning of the task the tube was centered on the middle point of the participant's ankles, had length of 114 cm and a fixed 5 cm diameter. Participants performed the task immediately after entering the virtual environment through the HMD. This measure provided the subject's baseline. The task was then repeated after each experimental condition.

According to the variant used in the experiment, the task returned one or two measures: Δ*TubeSize*, defined as the difference between the size of the tube set after each condition and its baseline value, provided a measure of changes in the perceived posture (i.e., the legs separation relative to the midline). Δ*TubeShift*, defines as the difference between the position of the tube set after each condition and its baseline value, provided instead an estimate for the perceived drift of the body midline, thus for *self-location*.

In the specific experimental design adopted in this study, we propose to assess *ownership* by looking at the effects of the strong visuo-proprioceptive coupling between the real and virtual body that is established during a FBOI (Maselli and Slater, 2013). The TT was then designed as tool for detecting such visuo-proprioceptive coupling: the experimental manipulations adopted included a change in posture of the virtual body whose legs spread apart while participants stayed still in the initial posture. Significantly positive values of Δ*TubeSize* will then be a proxy for an effective visuo-proprioceptive coupling between the real and virtual bodies, able to affect proprioception just via visual cues, and thus a proxy for the *ownership* illusion. Before applying this new method for testing the experimental hypotheses under study, the validity of the TT was tested in Experiment 1.

In Experiment 1, in which participants were asked to adjust only the tube's size, TT data were analyzed using One-Way repeated measures ANOVA with the single factor *condition* applied on the Δ*TubeSize*. In Experiment 2 the same analysis was applied separately to Δ*TubeSize* and Δ*TubeShift*. Pairwise comparisons between experimental conditions were then performed running paired *t*-tests.

#### The “Ankles Pointing Task” (APT)

This task was designed -through pilot studies- as an alternative to the TT for *ownership* assessments. As discussed in Section Discussion of the Results, we found that, while the TT is a sensitive test for *ownership* (via posture assessments) when only the length of the tube is controlled, it loses such sensitivity when combining *ownership* and *self-location* assessments and giving participants the possibility to change the tube's size and concurrently its lateral position. By comparing results from Experiments 1 and 2, it was indeed clear that tube's shifting option deteriorated the sensitivity in the feet separation estimate. For this reason we introduced the APT and designed it so that it could provide veridical estimates of the legs separation in a body centered reference frame, irrespectively of the visual perspective that participants were given in the different experimental conditions.

In this case participants were asked to point with the two hands the perceived location of the external ankles. Participants performed the task in complete darkness. They were instructed to first extend both arms out and then to progressively move them in, with the palms facing each other, until each hand would point the outer ankle on the correspondent side (see Movie SOM). The corresponding distance between the two hands was recorded through the tracking system (see Section Materials). As for the TT, the measure of interest was the changes in the hands' separation set after each condition with respect to its baseline value, Δ*HS.*

*As* Δ*HS* data from the APT Experiment 3 were not normally distributed, a Wilcoxon matched pairs test was adopted to compare the Δ*HS* distributions in the two experimental conditions.

#### Questionnaire

A 12-items questionnaire (reported in Table 1) was used in Experiments 2 and 3 to assess the subjective level and quality of the illusory experience. *Ownership* toward the virtual body was assessed explicitly (*my-body*) and implicitly through responses related to the legs spreading event (*legs-x*) as well as via the *clothing* statement that has been previously shown to positively correlate with the explicit subjective report of ownership illusion (Slater et al., 2010; Maselli and Slater, 2013). A further item was included to contrast the sense of *ownership* toward the virtual body with the sense of looking at another person (*s-else*). Changes in *self-location* were assessed with explicit items related to drifting sensations. Two items were included to validate the delivery of synchronous visuotactile stimulation. Each questionnaire item was scored at the end of each experimental block on a Likert scale from 1 (not at all) to 7 (completely agree) to express the level of agreement.

**Table 1:** **Questionnaire**.

**Item statement**	**Item tag**	**Illusory component**
I felt that the body I saw was my own body	*my-body*	Ownership
I felt that I was wearing different clothing than when I came to the laboratory	*clothing*	
I felt as if the body I saw belonged to someone else	*s-else*	
I felt as if I had two bodies	*two-bodies (C)*	
During the experiment, I felt as if my real body was drifting to the left	*drift-left*	Self-location
During the experiment, I felt as if my real body was drifting to the right	*drift-right*	
It seemed as if I was at two places at the same time	*two-places (C)*	
When I saw the virtual legs separating I felt as though my real legs were moving	*legs-moving*	Response to legs separation
I felt a weird sensation in my legs when I saw the virtual legs separating	*legs-weired*	
When I saw the virtual legs spreading apart, I felt as the instinct to spread apart my real legs	*legs-instict*	
It seemed as though the touch I felt was caused by the carton tube that I was seeing moving on the virtual body	*touch-tube*	Visuo-tactile implementation
It seemed as tough I felt the touch in the location where the carton tube touched the virtual legs	*touch-loc*	

Each questionnaire item was scored on a Likert scale from 1 (not at all) to 7 (completely agree) to express the level of agreement. The (C) label denotes control items.

For each item, scores given for different experimental conditions were compared pairwise via the non-parametric Wilcoxon matched pairs test.

### Experiments: designs and procedures

For the three experiments a within-subjects design was adopted. Each experimental condition was presented within one experimental block. Participants went through the different conditions in a counterbalanced order across participants, so that each condition was presented in the first experimental block the same number of times.

Participants wore a pair of comfortable trousers prepared so to easily arrange the four CCT vibrators held in direct contact with the skin by elastic bands. They sit on an armchair with their foot resting on a footstool and their hands on armrests, holding a joystick with their right hand (Figure 1). The separation between the feet was adjusted by the experiments at 18 cm. Participants were instructed to not make any movement, apart from head movements, for the whole duration of each experimental block. Before starting, the experimenter provided full instructions on how to perform the CCT and the TT or APT. All experiments started with an initial training session, preceding the experimental blocks: after a brief (about 10 s) exposure to the virtual environment, with a gender-matched virtual avatar displayed from a 1PP, participants were asked to perform the TT or APT; this initial measure was stored and used as the participant's baseline. Immediately afterwards, the virtual body appeared again and a CCT training session of 30 trials was initiated.

#### Experiment 1

**Figure 2:**
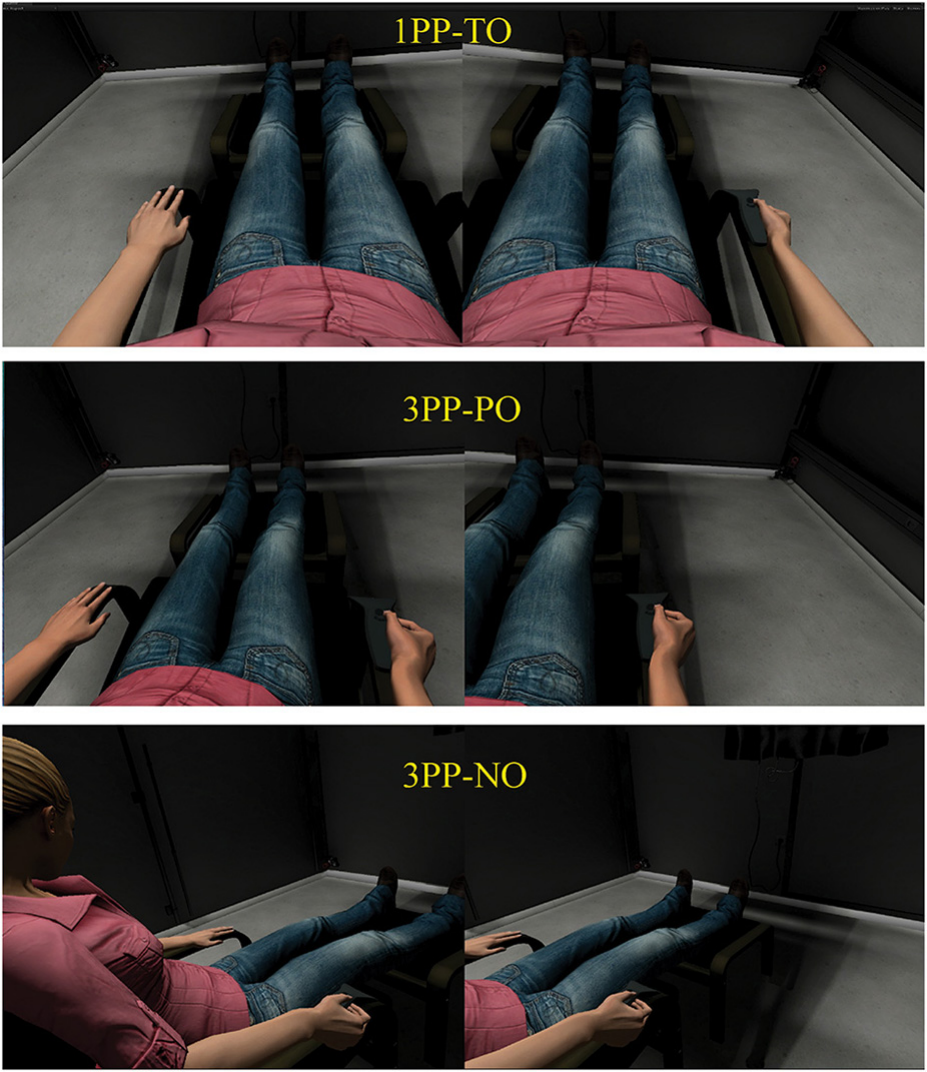
**Different visual perspectives**. According to the experimental conditions, participants had three different visual *perspectives* on the virtual body: a first person perspective with total overlap of the real and virtual bodies (*1PP-TO*); a third person perspective with partial overlap between the two bodies (*3PP-PO*); a third person perspective with no overlap between the two bodies (*3PP-NO*). In the *Body* and *Body+VT* conditions of Experiment 1, the visual perspective was the same as in 1PP-TO.

Three experimental conditions were included: in the *noBody* condition the virtual body was not displayed and participants saw an empty armchair when looking down. In the *Body* condition the virtual body was displayed to be spatially coincident with the physical body, i.e., from a first person perspective with total overlap between the real and virtual bodies (as in the upper panel of Figure 2). The *Body + VT* condition was the same as the *Body* one, but with the addition of continuous synchronous visuotactile stimulation via the tracked tube. In the *noBody* condition the visual perspective was the same as the first person perspective given in the two *Body* conditions, just the virtual body was not visible.

After completing the training phase, participants went through the three experimental blocks, each having an average duration of 10 min. In the initial phase (~1 min) participants were asked to first explore the virtual scene moving the head around and then to focus on the virtual body (*Body* conditions) or on the chair (*noBody*). Only in the *Body* + *VT*, synchronous VT stimulation was delivered: the experimenter slowly moved the tracked tube on both legs, simultaneously and symmetrically, from the feet to the thighs (see Movie in the SOM). The CCT was then started (~8 min). In the *Body* + *VT* condition, during the CCT the tube's stroking continued symmetrical and simultaneous on both legs but was restricted to the lower part of the calf and the feet (i.e., outside the region delimited by the CCT array). At CCT completion participants were again instructed to observe the scene and focus their attention on the virtual body or the chair (~1 min), as in the initial phase. In the two *Body* conditions, the avatar's legs moved apart after about 1 min, with the virtual feet reaching a distance of 55 cm in 3 s; in the *noBody* condition participants just looked at the empty chair for the same period of time. The “Tube Task” was then performed and terminated the experimental block: as the visual perspective was not manipulated, participants were instructed and only had the possibility to control the length of the tube, not its position. The HMD was removed and participants rested from 5 to 10 min before starting the next block.

The experiment was designed to test the response variables to be adopted in Experiments 2 and 3. The three main objectives were: (1) to show that the novel version of the CCT with vibrators and visual distracters arranged on the legs is equivalent to classical version (on the hands) and thus a valid tool for *self-location* assessments (see Section The Cross-Modal Congruency Task as a Measure of Self-Location); (2) to show that this novel version of CCT is still sensitive for detecting visuotactile integration processes on the self-body even when irrelevant visuotactile stimulation is concurrently delivered; (3) to validate the TT as a novel objective proxy for a full body *ownership* illusion.

In order to validate the first two points CCT's performances should reveal strong visuotactile interactions— i.e., a CCE significantly larger on the same side, with respect to opposite side— in both *Body* conditions. Note that we needed to validate point (2) in order to apply the CCT in the 3PP perspectives conditions of Experiments 2 and 3: in these conditions, in fact, it is necessary to keep providing visuotactile stimulation to sustain the full body illusion during the CCT, which is too long for a previously induced illusion to be sustained otherwise.

In the two *Body* conditions in fact, participants have a 1PP over a highly collocated realistic virtual body, a condition that has been shown to be sufficient for experiencing a high sense of ownership toward the virtual body (Maselli and Slater, 2013). We then expected that the TT would be able to corroborate the sense of *ownership* by revealing an increase in the perceived separation between the legs at the end of both *Body* condition with respect to the baseline value: because participants remained in static posture throughout each experimental block, such an increase would be driven by the just seeing the virtual legs spreading apart and indeed would reveal the strong visuoproprioceptive coupling between the real and the virtual body that is established during ownership illusions. In summary, in order to validate point (3), the distance between the feet estimated through the TT should be larger than the baseline value in both Body conditions and should not vary in the control *noBody* condition, in which no visual cues about body posture was available.

#### Experiment 2

Experiment 2 was designed to test the experimental hypotheses proposed in the introduction. The specific aim of the experiment was to show that it is possible to experience illusory changes in *self-location*, as in OBEs, with little if any illusion of *ownership* over the seen virtual body. At the same time, we expected that if *ownership* is induced over a dislocated body the perceived *self-location* should be strongly affected.

As in Experiment 1, we adopted a within-subjects design with three experimental conditions, in all of which the real and virtual bodies had the same posture. In *1PP-TO* condition, participants saw the virtual body from a 1PP with a total overlap (TO) between the real and virtual bodies. In the 3PP-PO condition the visual perspective was shifted laterally with respect to the virtual body, while keeping a partial overlap (PO) between the two; the lateral shift was set *ad-hoc* for each participant so that the right leg of the avatar coincided in external space with the left leg of the participant (average later shift of 25 cm). In the third condition, the visual perspective was shifted laterally 80 cm to the right of the virtual body, with no overlap (NO). The three different visual perspectives over the virtual body are shown in Figure 2. In all the three conditions synchronous visuotactile stimulation was delivered symmetrically on both legs via the tracked tube, in exactly the same way as in Experiment 1. Note to the *Body* + *VT* condition of Experiment 1 and the *1PP-TO* condition of Experiment 2 were exactly the same.

The procedure was the same as that described for Experiment 1. Participants went through all experimental blocks (one for each condition) after completing the training phase. Each block had an average duration of 10 min: the initial phase of tube's visuotactile stimulation (~1 min) was followed by the CCT (~8 min), a second phase of VT stimulation only (~1 min), the virtual legs spreading event (few seconds) and finally the TT. At the end of each block participants gave their scores to all questionnaire items while resting on the armchair.

The aim of the experiment was to test how *ownership* and *self-location* are modulated in FBI by shifting the visual perspective progressively away from the virtual body. Because we were interested in eliciting full body illusion we adopted the legs stroking as a trigger for the bodily illusions. The choice of the legs instead of, e.g., the chest, relays on several reasons: (i) first the legs and feet are visually prominent when looking down at our own body (if hands gesture is limited), particularly when laying in a resting posture; (ii) looking down at the chest requires a more targeted intention and is particularly uncomfortable in VR due to the HMD weight; (ii) stroking the legs allowed the stimulation of a wide portion of the whole body -from the feet to the thighs- and during the CCT to perform the stimulation while avoiding the region delimited by the CCT arrays; (iv) finally the legs separation could be easily manipulated without implying major changes in the overall resting posture.

#### Experiment 3

Experiment 3 was planned and designed as a follow up of Experiment 2, because of the failure of the TT in assessing changes self-posture when combined with self-location assessments (see discussion in Section The “Ankles Pointing Task” (APT) and Discussion of the Results). A within-subject design was adopted with two conditions: the *1PP-TO* and the *3PP-NO* of Experiment 2. The procedure was exactly the same of Experiment 2 with two differences. First the APT substituted the TT. Second, before starting each experimental block, participants were outfitted with headphones streaming white noise. This latter modification was introduced to avoid the potential inhibitory effect that conflicting visuo-auditory cues may have in the 3PP condition, in which a spatial mismatch was present between the sound produced by the real tube stroking the participant's legs and the virtual tube's location on the virtual body.

## Results

The analysis reported in this Section was performed excluding outliers from the distributions under test; outliers were identified via the schematic boxplot introduced by Tukey (1977). The degrees of freedom reported for each test give information about the number of outliers excluded. Requirements for normality were always checked with the Shapiro-Wilk test (Shapiro and Wilk, 1965) and reported with its *p*-value (p_SW_). We avoided the debated practice of correcting for multiple comparisons (Perneger, 1998), and instead report effect sizes together with the true *p*-value for each performed test.

The average RT, EP and IE extracted from the CCT data and averaged across participants are given in Table 2 as a function of *side, congruency*, and *condition*, for all the three experiments.

**Table 2:** **Experiments 1–3: Means (standard errors) from CCT performances**.

**Target-distactor congruence**	**Position of distracters**	**Reaction time (RT) [ms]**	**Error percentage (EP) [%]**	**Inverse IE = RT/(−EP) [ms]**
**EXPERIMENT 1**				
***NoBody***				
*Congruent*	*same*	600 (23)	1.0 (0.5)	606 (23)
	*different*	632 (22)	2.0 (0.9)	644 (20)
*Incongruent*	*same*	690 (28)	7.7 (1.2)	750 (33)
	*different*	680 (20)	3.7 (1.1)	707 (20)
***Body***				
*Congruent*	*same*	609 (29)	0.4 (0.2)	613 (31)
	*different*	650 (29)	1.0 (0.4)	656 (29)
*Incongruent*	*same*	723 (32)	4.8 (1.3)	764 (38)
	*different*	665 (28)	3.9 (0.5)	692 (30)
***Body + VT***				
*Congruent*	*same*	637 (32)	1.3 (0.6)	648 (35)
	*different*	682 (36)	3.6 (1.3)	707 (36)
*Incongruent*	*same*	738 (32)	9.2 (2.2)	823 (50)
	*different*	712 (31)	4.8 (1.4)	748 (30)
**EXPERIMENT 2**				
***1PP-TO***				
*Congruent*	*same*	638 (28)	2.2 (0.7)	653 (28)
	*different*	684 (30)	5.3 (1.1)	723 (57)
*Incongruent*	*same*	752 (30)	15.2 (3.3)	910 (31)
	*different*	713 (26)	7.3 (2.0)	776 (36)
***3PP-PO***				
*Congruent*	*same*	647 (19)	2.7 (1.0)	665 (20)
	*different*	694 (21)	4.0 (1.6)	726 (27)
*Incongruent*	*same*	751 (23)	13.8 (4.8)	977 (147)
	*different*	702 (20)	7.1 (2.0)	765 (35)
***3PP-NO***				
*Congruent*	*same*	656 (21)	4.7 (1.2)	691 (26)
	*different*	687 (23)	5.8 (1.1)	732 (58)
*Incongruent*	*same*	734 (23)	15.7 (3.6)	900 (28)
	*different*	745 (23)	12.7 (2.4)	860 (32)
**EXPERIMENT 3**				
***1PP-TO***				
*Congruent*	*same*	705 (33)	4.0 (1.2)	734 (38)
	*different*	735 (33)	6.8 (1.9)	789 (44)
*Incongruent*	*same*	818 (36)	8.2 (1.6)	887 (43)
	*different*	792 (32)	8.1 (2.0)	863 (44)
***3PP-NO***				
*Congruent*	*same*	724 (30)	3.5 (0.9)	753 (38)
	*different*	719 (28)	5.7 (1.6)	765 (36)
*Incongruent*	*same*	791 (34)	10.4 (2.1)	890 (53)
	*different*	783 (30)	7.1 (1.2)	841 (36)

*The reported values for reaction time, error percentage and inverse efficiency index are averages across participants. The variables are reported as a function of visual distracter side, congruency between vibrotactile and visual cues, and experimental condition*.

### Experiment 1

#### CCT

**Figure 3:**
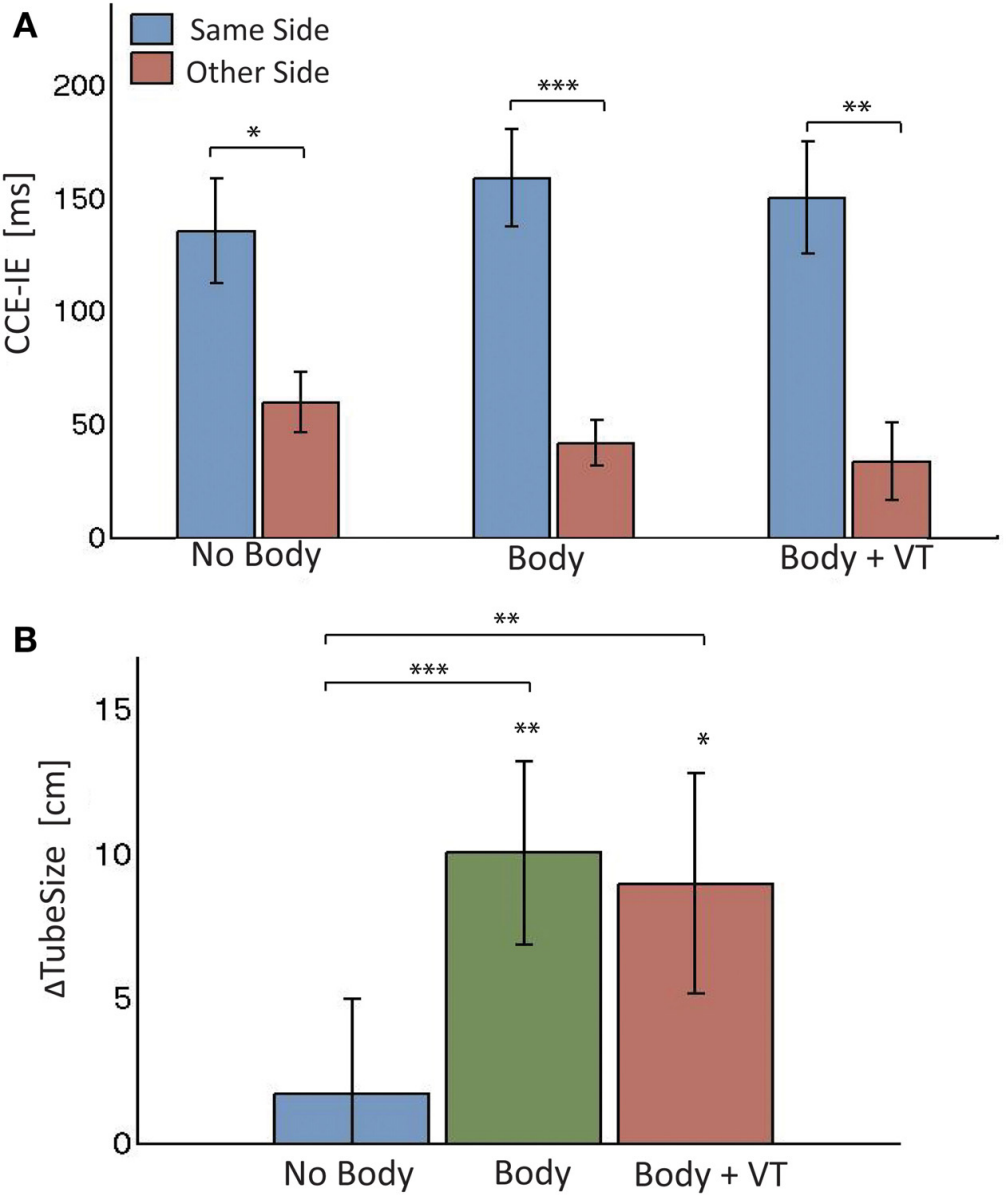
**Results from Experiment 1. (A)** Means and standard errors of the cross-modal congruency effect in inverse efficiency (CCE-IE) measured on the same side and on the opposite side, for the three experimental conditions (*noBody, Body*, and *Body+VT*). Ranges for the *p*-values from One-Way repeated measures ANOVA between same and other side are reported. **(B)** Differences (means ± standar errors) between the tube size estimation from the “*Tube Task*” (TT), perfomed after and before each of the experimental conditions: *noBody, Body*, and *Body+VT*. Positive values of Δ*TubeSize* indicate that participants experienced a recalibration of their perceived legs separation as a consequence of seeing the virtual legs spreading apart. Ranges for the *p*-values are reported for distributions with a mean significantly larger than zero (from one sample one-tailed *t*-test) and for paired comparisons across conditions (One-Way ANOVA). ^*^0.01 < *p* < 0.05, ^**^0.001 < *p* < 0.01, ^***^0.0001 < *p* < 0.001.

An average of 11% of responses were given for no-go trials across participants, none of which exceeded the 30%. Figure 3A shows the bar chart for the full body CCE-IE. In all conditions the effect is larger when the LD is on the same side with respect to opposite. Three-Way ANOVA with interactions showed a significant main effect of *congruency* [*F*_(1, 13)_ = 90.8, *p* < 0.0001, partial η^2^ = 0.39] and *condition* [*F*_(2, 13)_ = 8.7, *p* = 0.0003, partial η^2^ = 0.11], and a significant interaction term between *side* and *congruency* [*F*_(1, 13)_ = 24.4, *p* < 0.0001, partial η^2^ = 0.15]. Normality of the residuals was borderline (p_SW_ = 0.053).

Pairwise comparisons of the CCE-IE between same and opposite side showed a significant difference for all conditions [*noBody*: *F*_(1, 12)_ = 7.7, *p* = 0.017, partial η^2^ = 0.39; *Body*: *F*_(1, 12)_ = 48.1, *p* = 0.0001, partial η^2^ = 0.80; *Body + VT*: *F*_(1, 12)_ = 28.1, *p* = 0.0002, partial η^2^ = 0.72]. Residuals were normally distributed for all comparisons (p_SW_ > 0.3).

#### “Tube Task” (TT)

Figure 3B shows the bar plot for the Δ*TubeSize* distributions in the three conditions. All were normally distributed (p_SW_ > 0.85). Δ*TubeSize* is clearly larger than zero in the two *Body* conditions, as confirmed by one-sample one-tailed *t*-tests [*Body*: *t*_(14)_ = 3.17, *p* = 0.003, Cohen's *d* = 0.82; *Body + VT*: *t*_(14)_ = 2.36, *p* = 0.017, Cohen's *d* = 0.61]. This indicates a recalibration of the perceived posture. This was not the case for the *noBod*y (control) condition. This result shows that, when the perceived posture was not affected by visual cues from the virtual body, participants reliably replicated the initial baseline estimation of their legs separation. It is important to stress that, despite de within-subjects design, the body posture's estimate did not retain aftereffects or biases from previous exposure to the conditions in which the virtual body was seen changing its posture.

One-Way repeated ANOVA revealed a significant effect of *condition* on Δ*TubeSize* [*F*_(2, 14)_ = 6.5, *p* = 0.005, partial η^2^ = 0.26], with the residual being normally distributed (p_SW_ = 0.24). Pairwise comparisons revealed that Δ*TubeSize* was significantly larger for both *Body* conditions with respect to the *noBody* condition [*Body* vs. *noBody*: *t*_(14)_ = 3.4, *p* = 0.002, Cohen's *d* = 0.67; *Body + VT* vs. *noBody*: *t*_(14)_ = 2.37, *p* = 0.016, Cohen's *d* = 0.53]. No difference between the two *Body* conditions was found [*t*_(14)_ = 0.56, *p* = 0.58].

### Experiment 2

One subject was excluded from the analysis because he did not understand correctly the experiment instructions and another because the vibrators array stopped working during one of the conditions.

#### CCT

An average of 11.8% of responses were given for no-go trials across participants, none of which exceeded the 30%. Three-Way ANOVA with interactions revealed a significant main effect of *congruency* [*F*_(1, 12)_ = 68.4, *p* < 0.0001, partial η^2^ = 0.34], a significant interaction term between *side* and *congruency* [*F*_(1, 12)_ = 20.0, *p* < 0.0001, partial η^2^ = 0.13], and a trend for interaction of *side, congruency* and *condition* [*F*_(1, 12)_ = 2.7, *p* = 0.071, partial η^2^ = 0.04]. The requirement for normality of the residuals was satisfied (p_SW_ = 0.08).

**Figure 4:**
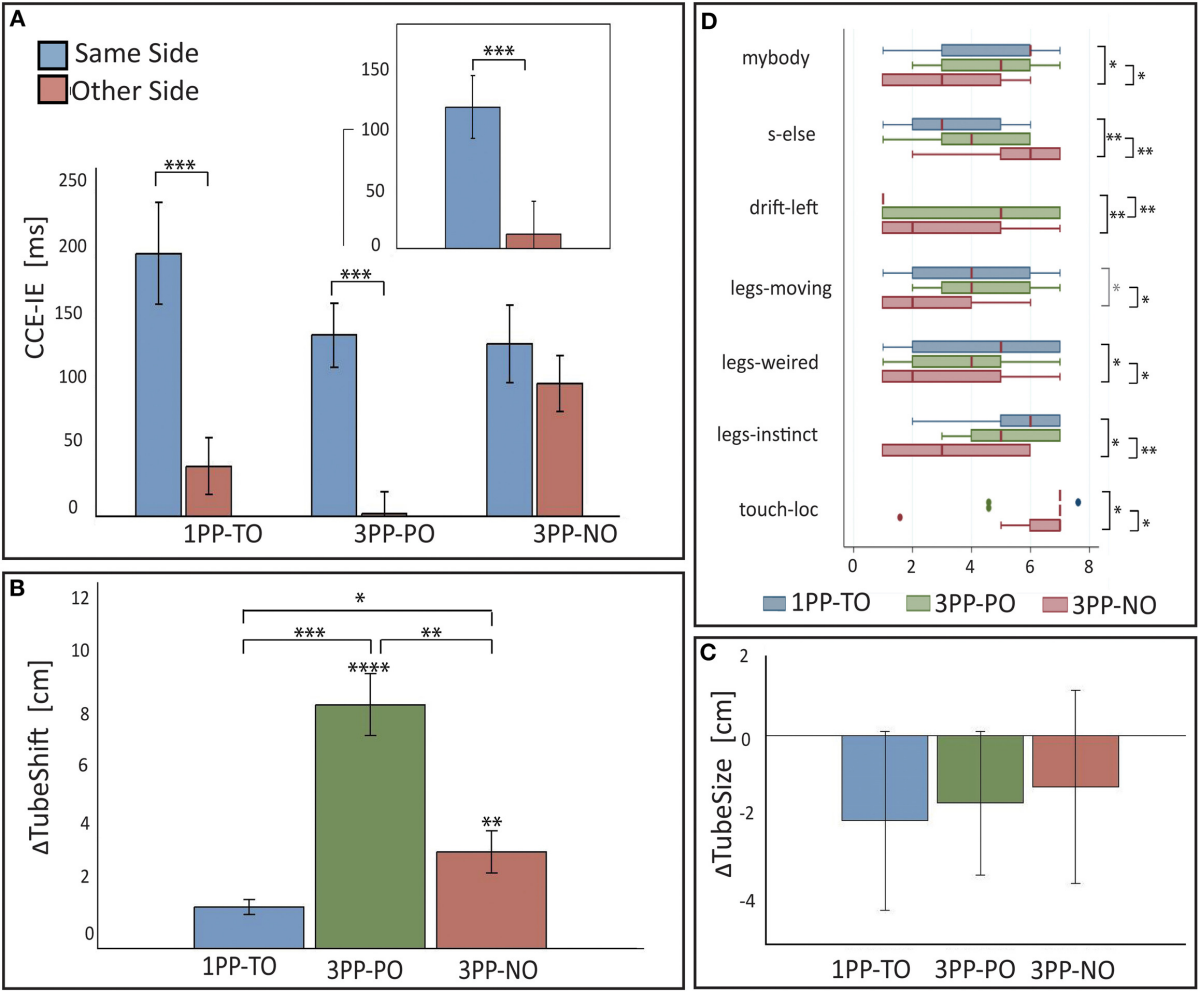
**Results from Experiment 2. (A)** Means and standard errors of the cross-modal congruency effect in inverse efficiency (CCE-IE) measured on the same side and on the opposite side, for the three experimental conditions (*1PP-TO, 3PP-PO* and *3PP-NO*). The inset shows the same for the *3PP-PO* subset of trials in which the target vibration was on the real left leg that overlapped in external space with the right virtual leg. Ranges for the *p*-values from One-Way repeated measures ANOVA between same and other side are reported. (**B)** Differences (means ± standar errors) between the tube lateral position set in the *Tube Task* (TT) after and before each experimental condition. Positive values of *ΔTubeShift* indicate that participants experienced a recalibration of their *self-location* to the left, which is toward the virtual body. Ranges for the *p*-values are reported for distributions with a mean significantly larger than zero (from one sample *t*-test) and for paired comparisons across conditions (One-Way ANOVA). **(C)** Differences (means ± standar errors) between the tube size estimation from the *Tube Task* (TT), perfomed after and before each experimental condition. The Δ*TubeSize* distributions match the zero mean distribution in all the three conditions. **(D)** Box plots showing the comparison of questionnaire data across conditions: only items for which significant differences among conditions have been found are shown. Ranges for the *p*-values from matched pairs Wilcoxon tests are reported. ^*^0.01 < *p* < 0.05, ^**^0.001 < *p* < 0.01, ^***^0.0001 < *p* < 0.001, ^****^0 < *p* < 0.0001.

Pairwise comparisons (Figure 4A) revealed a significantly higher CCE-IE on the same side with respect to opposite in *1PP-TO* [*F*_(1, 12)_ = 27.7, *p* = 0.0002, partial η^2^ = 0.70] and *3PP-PO* [*F*_(1, 12)_ = 74.2, *p* < 0.0001, partial η^2^ = 0.86], but not in *3PP-NO* [*F*_(1, 12)_ = 1.13, *p* = 0.31]. Residuals were normally distributed for all comparisons (p_SW_ > 0.26). Because we expected a significant difference between the CCE on same and other side also in *3PP-NO*, we repeated the test with the CCE computed for the RT data (CCE-RT). In this case we found CCE-RT to be higher in the same than in the other side [*F*_(1, 14)_ = 4.57, *p* = 0.0507, η^2^ = 0.051, p_SW_ = 0.17], in line with our expectations.

It was interesting to specifically inspect performance on the *3PP-PO* trials with the target vibration on the left leg of the participant. In fact, for these trials, the light distracters on the “opposite side” were located, in external space, in exactly the same positions of the vibrators (the virtual right leg overlapped in external space with the participant's left leg), while those on the “same side” were spatially incongruent. Despite this, the average CCE-IE was much higher on the same side than on the opposite (see inset in Figure 4A). A significant difference between same and opposite side was detected in this subsample with a matched-paired Wilcoxon test (z_13_ = 3.1, p_13_ = 0.002, PS_dep_ = 0.92), as when running an One-Way ANOVA the resulting residuals were not normally distributed.

#### “Tube Task”

Due to technical recording problems, the TT data were missing from three subjects of the 15 subjects under analysis. The Shapiro Wilk test revealed normal distributions for both Δ*TubeSize* and Δ*TubeShift* (p_SW_ > 0.24). Two outliers (one in *1PP-TO* and one in *3PP-NO*) were found and excluded from the analysis.

Figure 4B shows the bar chart for the Δ*TubeShift* distributions. For all conditions the distributions were normal (p_SW_ > 0.20). One-sample one-tailed *t*-test analysis revealed a distribution mean significantly larger than zero for both the *3PP-PO* [*t*_(11)_ = 7.59, *p* < 0.0001, Cohen's *d* = 2.19] and *3PP-NO* [*t*_(10)_ = 2.8, *p* = 0.0097, *d* = 0.84], but not for *1PP-TO* [*t*_(10)_ = 1.5, *p* = 0.086, *d* = 0.44].

One-Way repeated ANOVA revealed a significant effect of condition on Δ*TubeShift* [*F*_(2, 9)_ = 13.66, *p* = 0.0002, partial η^2^ = 0.60], with normally distributed residuals (p_SW_ = 0.99). Pairwise comparisons revealed that Δ*TubeShift* was significantly larger in both *3PP* conditions with respect to *1PP-TO* [*3PP-PO* vs. *1PP-TO*: *t*_(10)_ = 5.4, *p* = 0.0002, Cohen's *d* = 2.62; *3PP-NO* vs. *1PP-TO*: *t*_(9)_ = 2.2, *p* = 0.029, *d* = 0.92]. Interestingly, Δ*TubeShift* in the *3PP-PO* condition was found to be significantly larger than in *3PP-NO* [*t*_(10)_ = 3.4, *p* = 0.0036, Cohen's *d* = 1.79].

The Δ*TubeSize* values were normally distributed in all conditions (p_SW_ > 0.24). Contrary to our expectations the TT did not detect significant changes in the perceived posture for any of the conditions (see Figure 4C). One-sample *t*-test performed on the three distributions revealed indeed no difference from the zero-mean distribution (*p* > 0.29). One-Way repeated ANOVA also revealed no significant difference between conditions [*F*_(2, 11)_ = 1.73, *p* = 0.2].

This unexpected outcome may be due to the interaction between the tube size adjustment and the additional lateral shift. In fact the *1PP-TO* condition in this experiment is exactly the same as the *Body + VT* condition in Experiment 1, with the only difference being instructions given for the TT. The fact that in Experiment 1 the distribution of Δ*TubeSize* has a mean significantly larger than zero, while in Experiment 2 its mean is statistically equal to zero suggests that having the possibility to shift the tube laterally, and actually doing it, has a detrimental effect on the TT's measure of perceived body posture. The only difference between the two groups was in fact that in the latter participants had the possibility to shift the tube's laterally a part from adjusting its size.

#### Questionnaire

Median values and interquartile ranges are reported in Table 3 for all items. The results from matched pairs Wilcoxon tests are given in Table 4 (items for which none of the three comparisons was significant were omitted). Effect sizes are given in terms of the probability of superiority of dependent measures, PS_dep_, defined as the probability that the score from the condition that most frequently has the higher score will be greater than the score from the condition that most frequently has the lower score (Grissom and Kim, 2012). The table highlights all significant comparisons as shaded cells. Boxplot corresponding to the significant comparisons are shown in Figure 4D.

**Table 3:** **Experiment 2: Medians and interquartile ranges of the questionnaire scores**.

	**1PP-TO**		**3PP-PO**		**3PP-NO**	
**Item**	**Median**	**IQR**	**Median**	**IQR**	**Median**	**IQR**
*my-body*	6	3	5	3	3	4
*clothing*	6	4	5	5	4	3
*s-else*	3	3	4	3	6	2
*two-bodies*	2	2	3	4	4	4
*drift-left*	1	0	5	6	2	4
*drift-right*	1	0	1	4	1	1
*two-places*	3	2	3	4	3	4
*legs-moving*	4	4	4	3	2	3
*legs-weird*	5	5	4	3	2	4
*legs-instinct*	6	2	5	3	3	5
*touch-tube*	7	1	7	1	7	1
*touch-loc*	7	0	7	0	7	1

**Table 4:**
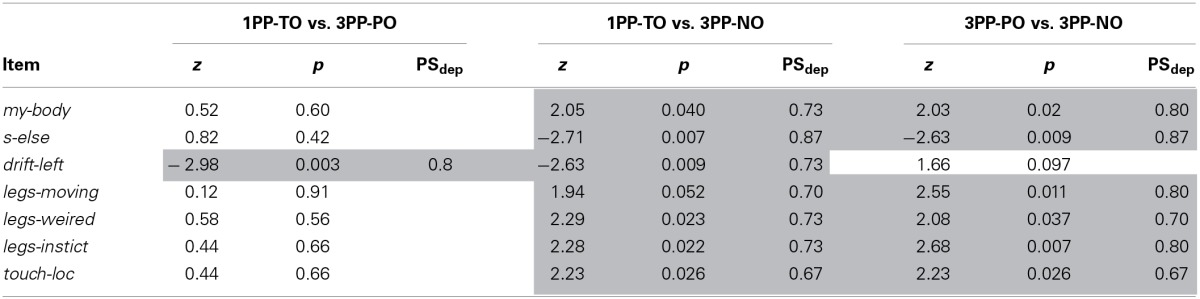
**Experiment 2: Paired comparisons of the questionnaire scores**.

Reported p-values are from matched pairs Wilcoxon tests; effect size is reported in terms of the probability of superiority of dependent measures (PS_dep_). Only results from comparisons revealing a significant difference across conditions are reported. Shaded cells highlight comparisons that revealed a significant difference across conditions.

### Experiment 3

#### CCT

**Figure 5:**
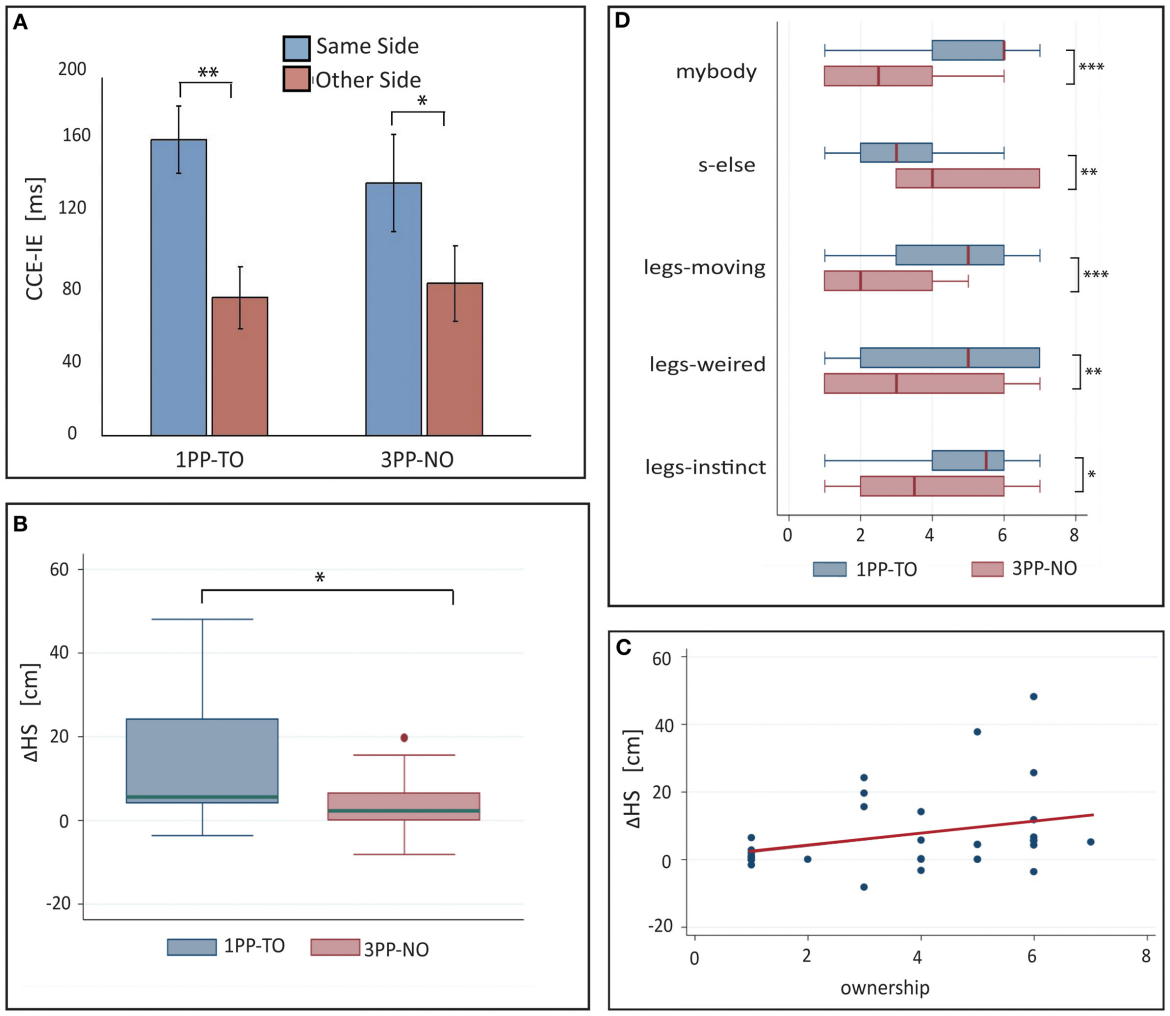
**Results from Experiment 3. (A)** Means and standard errors of the cross-modal congruency effect in inverse efficiency (CCE-IE) measured on the same side and on the opposite side, for the two experimental conditions (*1PP-TO* and *3PP-NO*). Ranges for the *p*-values from One-Way repeated measures ANOVA between same and other side are reported. **(B)** Box plot of the differences between the hands separation measured in the “*Ankles Pointing Task*,” after and before each experimental condition. Positive values of Δ*HS* indicate that participants experienced a recalibration of their perceived legs separation as a consequence of solely seeing the virtual legs spreading apart. Δ*HS* values were significantly larger in *1PP-TO* than in *3PP-NO*, showing that a proprioceptive recalibration occurred in the first condition but not in the latter. The *p*-value from matched pairs Wilcoxon test is reported. **(C)** Δ*HS* values are plotted against the scores to the *mybody* questionnaire item. A strong statistical trend (*p* = 0.055) for a positive correlation between the two variables was found. **(D)** Box plots showing the comparison of questionnaire data across conditions: only items for which significant differences among conditions have been found are shown. Ranges for the *p*-values from matched pairs Wilcoxon tests are reported. ^*^0.01 < *p* < 0.05, ^**^0.001 < *p* < 0.01, ^***^0.0001 < *p* < 0.001.

Three-Way ANOVA with interactions revealed a significant main effect of *congruency* [*F*_(1, 13)_ = 94.4.0, *p* < 0.0001, partial η^2^ = 0.51] and a significant interaction between *side* and *congruency* [*F*_(1, 13)_ = 6.3, *p* = 0.014, partial η^2^ = 0.06]. Importantly, the three-way interaction between *congruency, side* and *condition* was not significant, indicating that visuotactile interactions were comparable in both conditions. The requirement for normality of the residuals was satisfied (p_SW_ = 0.97). Pairwise comparisons between CCE-IE on same side and opposite side (shown in Figure 5A) revealed a significant difference in both the *1PP-TO* [*F*_(1, 15)_ = 15.7, *p* = 0.0012, partial η^2^ = 0.51] and the *3PP-NO* [*F*_(1, 15)_ = 5.79, *p* = 0.029, partial η^2^ = 0.28]. Residuals were normally distributed (p_SW_ > 0.19).

#### “Ankles Pointing Task” (APT)

Data from two subjects were missing due to recording problems. Another two data sets were discharged because of the participant having misinterpreted the task instructions. A total of 15 subjects were included in the analysis. It is notable that the Δ*HS* estimates from these two subjects were “extreme” outliers (Tukey, 1977) in the Δ*HS* distribution from *3PP-NO*.

The Δ*HS* distribution extracted from the APT was not normal, but resembled a bimodal distribution with one cluster around zero and one another at positive values. This was particularly the case for the *1PP-TO* distribution. The cluster around zero was probably a biased from participants that based their measure on the knowledge that their feet did not mov, as explicitly reported by one of the two excluded participants.

A matched-pairs Wilcoxon rank test revealed a significant difference between the two experimental conditions (*z*14 = 2.42, *p*14 = 0.016, PS_dep_ = 0.71), indicating that participants perceived a significantly larger distance between their own ankles in the 1PP-TO than in the 3PP-NO. The box plot of the two distributions is shown in Figure 5B.

#### Questionnaire

Questionnaire results are summarized in Table 5 and shown in Figure 5D. The results are consistent with those from Experiment 2, apart from the *drift-left* item. Scores were in fact significantly higher in *1PP-TO* than in *3PP-NO* for the items *my-body* (*z* = 3.27, *p* = 0.001, PS_dep_ = 0.89) and the three items assessing the response to the legs separation event (*legs-moving*: *z* = 3.18, *p* = 0.001, PS_dep_ = 0.86; *legs-weird*: *z* = 2.65, *p* = 0.008, PS_dep_ = 0.78; *legs-instinct*: *z* = 2.29, *p* = 0.022, PS_dep_ = 0.72). On the other hand, scores *s-else* were significantly higher in *3PP-NO* than in *1PP-TO* (*z* = 2.9, *p* = 0.004, PS_dep_ = 0.78). Differently from Experiment 2, no significant difference was found for *drift-left*, although a weak trend for scores to be higher in *3PP-NO* than in *1PP-TO* was found (z = 1.52, *p* = 0.13). This was due to the extremely low scores given to this item in the *3PP-TO* condition across all participants, which was not the case in Experiment 2.

**Table 5:**
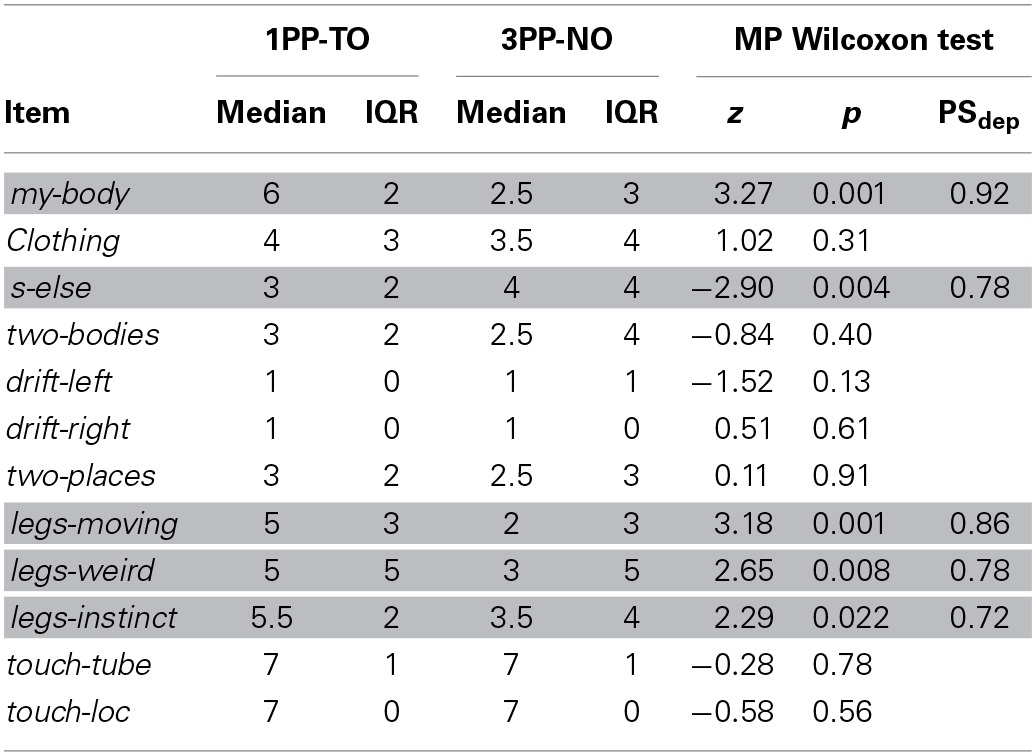
**Experiment 3: Medians, interquartile ranges and paired comparisons of the questionnaire scores**.

Reported p-values are from matched pairs Wilcoxon tests and effect size is reported in terms of the probability of superiority of dependent measures (PS_dep_). Shaded cells highlight comparisons that revealed a significant difference across conditions.

#### Correlation analysis

We tested the correlation between the level of ownership as assessed from questionnaire scores to the *mybody* item and the changes in the perceived legs separation measured with the APT (Δ*HS*). The Spearman's rho was showing a close to statistical significant positive correlation between the two measures (ρ = 0.34, *p* = 0.055), also shown in the scatter plot in Figure 5C. It is remarkable that despite the cognitive bias discussed above (participants knew that their feet did not move), which introduces extra variance in the APT data hindering correlation analysis, a positive correlation was found. This result further corroborates the validity of APT method as measure for ownership illusions.

## Discussion of the results

### Experiment 1

Results from Experiment 1 validated the methods used in the study. It was shown that the new proposed version of CCT, with the LEDs/vibrators arranged on the legs, is a valid tool to detect effective integration of visuotactile (VT) bodily signals, and thus to detect changes in the perceived *self-location* during full body illusions (see discussion in Section Materials and Methods). The CCT revealed in fact effective visuotactile interactions in both the *Body* conditions, in which participants experienced a fully collocated virtual body from a 1PP, a condition shown to be sufficient for eliciting a FBOI (Maselli and Slater, 2013). Importantly, the effective SOA was 20 ms, so that effective VT interactions can be primarily attributed to effective VT integration (Shore et al., 2006). In a similar setup, Aspell et al. (2009) had instead to increase the SOA to 233 ms in order to detect effective VT interactions when additional visuotactile stimulation was delivered during the CCT; however, as a drawback, the observed interaction was mainly dominated by exogenous attention rather than MSI (Shore et al., 2006). This difference may be due to the fact that, in our study, the visuotactile stimulation was delivered outside the region delimited by the CCT lights/vibrators array.

Interestingly, effective VT integration was detected also in the *noBody* condition. In previous studies, a similar condition was implemented, but with the array of visual distracters shifted with respect to the vibrators: in the “rubber hands absent” condition of Pavani et al. (2000) the vibrators were located on the unseen hands and the LEDs array was shifted 20 cm upward; similarly, in the “body not visible” condition of Aspell et al. (2009) the vibrators, located on the back while the distracters, were seen in the far extra-personal space. In both studies no significant visuotactile interaction was detected when the body was not visible. A significant CCE effect has instead been reported for an object condition in Salomon et al. (2012); however, as suggested by the authors, it is not straightforward to interpret this results because of the experimental design adopted, in which “object” trials where intermixed at a fast pace with “body” trials (Salomon et al., 2012). The fact that we did find evidence for visuotactile interaction also when the body is not visible is most probably due to the fact that LED distracters and vibrators were in spatial register. The result shows that the spatial information about the body provided solely by proprioception is sufficient to have a robust stored localization of body parts that contribute to the multisensory integration of visual and tactile information. This is consistent with the previous findings of the CCE modulation associated with various degree of visuo-proprioceptive spatial mismatch (Spence et al., 2008).

Experiment 1 further validated the TT/APT as novel tools for objective assessments of full body ownership illusions. The TT data showed that in the two *Body* conditions (in which *ownership* was experienced toward the virtual body) after seeing the virtual body moving the feet apart and getting into a new posture, participants estimated a larger distance between their feet even if the physical body did not move at all. This outcome relays, as expected, on the strong coupling of visual and proprioceptive cues from the virtual and real bodies that is established during an *ownership* illusion (see Section 4.4 in Maselli and Slater, 2013). In this respect, the validation of the TT as a tool to assess full body *ownership* through assessments of illusory changes in posture driven by movements of the virtual body only extends to the APT. In fact, in the latter differs from the TT only in the way in which participants indicated the perceived distance among their feet, which was design *ad-hoc* to provide estimates of the legs separation in a body centered reference frame. The validity of the APT as a proxy for the FBOI was further corroborated by the positive correlation found between ownership measures from questionnaire (scores to the *mybody* item) and the illusory change in body postures measured with the APT (Δ*HS*), found in Experiment 3.

### Experiments 2 and 3

The outcome of Experiments 2 and 3 supports our main experimental hypotheses. We first discuss the set of results that show how *ownership* and *self-location* can be manipulated independently from each other, during FBOI and OBE illusion respectively. We do this by comparing data from the *1PP-NO* and *3PP-NO* conditions. We then discuss results that show how simultaneous changes in *ownership* and *self-location* can be induced, and do interact, when having a third person visual perspective of a virtual body that partially overlaps with the physical body, as in the *3PP-PO* condition.

#### FBOI and OBE can occur independently of each other

As expected we found evidence for FBOI to occur in the *1PP-TO* but not in the *3PP-NO* in both questionnaire and APT data. Scores to the *my-body* item, as well as to all the three *legs* items, were significantly higher in *1PP-TO* than in *3PP-NO*, showing that *ownership* toward the virtual body was experienced consistently across participants, only in the *1PP-TO* condition. The APT data from Experiment 3 corroborated this finding. A significantly larger change in the perceived feet separation was in fact found in the *1PP-TO* condition with respect to *3PP-NO*. This showed that a tight coupling between vision and proprioception, characteristic of ownership illusions, is established in the *1PP-TO* but not in the *3PP-NO* condition. In both Experiments 2 and 3, scores to the *s-else* item were significantly higher in *3PP-NO* than in *1PP-TO*. This complementary result shows that the virtual body seen in the far extra-personal space was experienced as the body of another person rather that as one's own body, supporting the finding that no *ownership* is generally experienced toward the virtual body seen in the far extra-personal space. Despite this, both TT and CCT performances consistently show that actual changes in the perceived *self-location* took place in *3PP-NO*. The distribution of *ΔTubeShift* in 3PP was found to be significantly larger than zero (indicating a perceived lateral shift in the perceived position of the whole body) and significantly larger than the *ΔTubeShift* measured in 1PP-TO (which was compatible with no lateral shift). This finding is consistent with results from previous studies of experimentally induced OBEs, which measured similar shifts of the perceived *self-location* toward the virtual body using methods akin, e.g., the walking task (Lenggenhager et al., 2007) and the mental ball dropping task (Lenggenhager et al., 2009; Ionta et al., 2011). Notwithstanding the evidence provided by questionnaire and TT data for an altered *self-location* in 3PP-NO, the CCT data from Experiment 2 in this condition did not provide clear evidence for integration of visual cues on the virtual body and tactile cues from the correspondent body locations on the physical body (the CCE-IE was not significantly dependent on *side*). This was indeed contrary to our expectation. However, it is worth noting that a strong trend (*p* = 0.052) for a significant cross-modal interaction was found in the CCE-RT data (CCE measured on response times). Additionally, only a weak trend for a significant difference across conditions was found in the CCE-IE ANOVA (*p* = 0.07), which shows that the data do not provide clear evidence for CCT performance to be different in *3PP-NO* than in the other two conditions, where a strong VT interaction was detected. The lack of a significant CCE-IE effect in *3PP-NO* may be due to fact that visual distracters were far on the left side of the visual field, with a loss of sensitivity. In this case a larger sample would be needed. Also, conflictive visuo-auditory cues in *3PP-NO* (from the tube stroking) may have acted as additional confounders making the task more difficult, and/or contributing negatively to the OBE illusion itself. These speculations are supported by CCT performances in Experiment 3, in which white noise was introduced to mask visuo-auditory incongruences on a larger sample of participants. A significant difference in the CCE-IE across same and different sides was indeed found in both *1PP-TO* and *3PP-NO*. This indicates that, during an OBE illusion, visual events experienced on the virtual body and tactile events experienced on the real body are integrated despite the dramatic spatial separation between them. It is important to notice that the feeling of another person to be present that may arise in our 3PP-NO condition (and generally in all experimental setups inducing full body illusion from a 3PP), may induce social mechanisms that affects self-processing (Serino et al., 2008), and in turns hinder the FBI. In any case, joint evidence from questionnaire (*drift-left item*), the CCT and the TT in the 3PP-NO condition speaks in favor of an actual shift of the perceived *self-location* toward the virtual body.

Summarizing, results from Experiments 2 and 3 provided the first experimental evidence that OBE and FBOI are two diverse perceptual illusions affecting different aspect of body self-consciousness. This was established by showing that both could occur independently of each other and that the two manifest themselves in different perceptual phenomena. In the general discussion we provide arguments for the different neural correlates that may be predominantly involved in each of the two illusions.

#### FBOI during OBEs: evidence for a strong interaction of ownership and self-location

Experiment 2 provided novel evidence on how the sense of *ownership* and the sense of *self-location* influence each other when they are concurrently manipulated. All data collected show that this was the case for the *3PP-PO* condition, in which the visual perspective was dislocated from the eyes of the virtual avatar (3PP) while preserving a substantial overlap between the two bodies: participants in this condition experienced a strong FBOI over the virtual body with a concurrent significant change in the perceived *self-location*.

Apart from the *drift-left* item, questionnaire scores in this condition were statistically equal to those given in *1PP-TO*. At the same time, the comparison between this condition and the *3PP-NO* condition revealed the same significant differences found in the *1PP-TO* vs. *3PP-NO* comparison. This indicated that a strong FBOI occurred similarly in both *1PP-TO* and *3PP-PO*. On the other hand, TT data showed that participants experienced a strong drift in the perceived *self-location*. Furthermore, the drift was significantly stronger than that experienced in *3PP-NO*, despite the fact that the actual displacement among the virtual and real bodies was much greater in the latter.

Analogously, the CCT data from the *3PP-PO* condition support a significant shift in the perceived *self-location*. The highly significant cross-modal interaction found in *3PP-PO* clearly showed that vibrotactile cues were indeed processed in an updated spatial register in which tactile events on the real body and visual events on the virtual body are associated according to a precise bodily-based topological correspondence. This result is even more remarkable when considering that, in this condition, the vibrotactile cues on the real left leg of participants were exactly collocated with the LEDs on the right leg of the virtual body, so that if no shift in the perceived *self-location* occurred we should have observed a strong interaction among these visuotactile cues, which was instead not observed (see inset in Figure 4B).

Summarizing, the experimental manipulation in the *3PP-PO* condition induced concurrent changes in *ownership* and *self-location*. Most importantly, the latter was much stronger than the analogous change experienced in the *3PP-NO* condition, indicating a strong interaction between *ownership* and *self-location*. Despite the fact that the two can be dissociated from each other, it seems that the sense of *ownership* experienced over a dislocated virtual body can importantly boost the experienced drift in the perceived *self-location*.

## General discussion

The experimental study here presented provides direct experimental evidence that the sense of *ownership* and the sense of *self-location* can be selectively manipulated in full body illusions. It was shown that it is possible to experience illusory changes in the perceived *self-location* during OBEs when no sense of *ownership* is experienced toward the virtual body. On the other during FBOI experienced toward a fully collocated virtual body, the perceived *self-location* was (obviously) not altered, as no conflict in the location of the two bodies was present.

The fact that OBE illusions can occur without changes in the sense of *ownership* and that OBE and FBOI are indeed two different perceptual illusions was previously suggested (Petkova et al., 2011b; Maselli and Slater, 2013), but to date it was not supported by experimental data.

Previous studies on OBE illusions have been primarily designed to manipulate and measure illusory changes in *self-location*. Despite this OBE illusions are often considered and discussed as belonging to the wider class of full body *ownership* illusions (e.g., Blanke, 2012; Limanowski et al., 2013). Having here shown that FBOI and OBE are different perceptual illusions is thus particularly important, because the lack of a clear distinction between the two illusions often resulted in interpreting observed changes in *self-location* as evidence for a sense *ownership* experienced toward a body seen in the far extra-personal space (Lenggenhager et al., 2007; Aspell et al., 2012).

As a second important outcome, the present study showed that when seeing a virtual body that overlaps only partially with the space occupied by the physical body, a strong FBOI is experience together with a significant shift in the perceived *self-location* toward the virtual body. This is indeed the first explicit experimental evidence for a FBOI experienced while having a third person visual perspective over a fake full body. Our data further show a positive interaction between the two components of body self-consciousness: in this “partial overlap” configuration, the experienced shift in *self-location* is in fact significantly larger than the one experienced in an OBE illusion. It seems then that, when both *ownership* and *self-location* are affected during a full body illusion, it is the sense of *ownership* that drives the sense of *self-location* toward the owned body.

Although our study does not provide any direct evidence for the existence of distinct neuronal correlates for FBOI and OBEs, in the following we propose plausible candidates for the sets of neuronal structures that are likely involved in the two illusions.

### On the neuronal correlates of experimentally induced OBEs and peripersonal space

It has been recently proposed (Blanke, 2012) that the best neural candidates for encoding the perceived shift in *self-location* experienced during experimentally induced OBEs are the populations of bimodal visuotactile (VT) neurons hosted in cortical and subcortical areas of the primates brain.

Bimodal visuotactile populations are characterized by tactile receptive fields that can cover small patches or large portions of the body surface, and by visual receptive fields (vRF) anchored to the body part mapped in the tactile modality (Graziano and Gross, 1993; Duhamel et al., 1998; Brozzoli et al., 2012; Huang et al., 2012). For these characteristic properties bimodal VT populations have been suggested to encode the peripersonal space (Fogassi et al., 1996): through their vRFs these populations define in fact the region of space surrounding the body in which visual stimuli may trigger somatic sensations. The main functional role of this dynamic representation of the external space in body-parts centered coordinates is to guide movements for defensive purposes and for efficient interaction with external objects (Graziano and Cooke, 2006; Sereno and Huang, 2014).

Results from single cell recording in the monkey have further shown that the vRF of bimodal visuotactile neurons is highly plastic and can expand after the active manipulation of tools (Iriki et al., 1996). This groundbreaking result provided the first robust experimental evidence that the peripersonal space is highly plastic and can be reshaped in relatively short time scales. The plasticity of bimodal VT neurons has been then advocated as the mechanism that allows healthy humans to embody external objects, like tools, in their body schema (Maravita and Iriki, 2004).

As discussed thoroughly in Section The Cross-Modal Congruency Task as a Measure of Self-location, the cross-modal congruency task (CCT) has been shown to be a powerful psychophysical test to detect dynamic modulations of the peripersonal space during active tool use (Maravita et al., 2002), as well as during a rubber hand illusion (Pavani et al., 2000; Zopf et al., 2010). In line with these results, CCT performances in our study showed that, during experimentally induced OBEs, an actual extension (or shift) in the vRF of proximal visuotactile neurons occurs. This modulation can be interpreted as a modulation of the peripersonal space likely triggered by VT neural populations that maps large portions of the body, such as the legs. It is worth noting that neuronal populations with such properties have been identified in the human posterior parietal cortex (PPC) (Huang et al., 2012), and that the PPC has be shown to play a crucial role in mapping bodily signal into external space (Azañón et al., 2010).

In line with the original proposal from Blanke (2012), we argue that the dynamical changes in the vRF of visuotactile neurons with large receptive field on the body surface, is responsible for the perceived changes in *self-location*. We further argue that the same mechanisms may be regarded as the minimum common denominator of all OBEs. This proposal is not in conflict with the well-known implication of the TPJ in OBEs, which has been revealed in lesion analysis of patients suffering from recurrent OBEs (Blanke and Arzy, 2005), in electro-stimulation studies showing that OBEs can be systematically induced by direct stimulation of the TPJ (Blanke et al., 2002), and with fMRI performed during experimentally induced OBEs (Ionta et al., 2011). The latter study showed in fact that activity in the TPJ seems to encode the perceived elevation of the whole body -rather than its position in extra-personal space- and thus it is plausible that the TPJ implication in OBEs occurs at higher level in the hierarchical processing of sensory signals, in which a further integration is operated to combine visuotactile cues with vestibular and auditory information (Lopez et al., 2008).

### Different representations for the sense of ownership and self-location

Our study has shown that OBEs can occur with or without an associated *ownership* illusion. This implies that the dynamic changes in the peripersonal space associated with OBEs may be coupled or not with the activity of a larger and more complex network of neuronal populations that give rise to the sense of *ownership* during FBOI. Imaging studies have shown that the illusion of ownership for both body parts (RHI) and full bodies involves a complex network of brain areas that is still not well established and controversial. Depending on the experimental design and the imaging technique adopted, the ownership illusion has been found to correlate with activity in several brain areas: ventral premotor cortex (vPMc) and posterior parietal cortex (PPc) (Ehrsson et al., 2004; Petkova et al., 2011a), primary somatosensory cortex (Limanowski et al., 2013; Shokur et al., 2013), the extrastriate body area (EBA) (Limanowski et al., 2013); and, at subcortical level, the insula (Tsakiris et al., 2007; Limanowski et al., 2013). Recent theoretical accounts for body ownership have stressed the role of multimodal hierarchical processing of sensory and motor signals from the body (e.g., Hohwy, 2007; Ehrsson, 2011; Moseley et al., 2012; Limanowski and Blankenburg, 2013). Although a review of these works is beyond the scope of the present work, we want to stress here the potential contribution that our results may bring about.

The present study has highlighted, in particular, the fundamental role of visuo-proprioceptive correlations in driving FBOI. In a previous study we showed how congruent visuo-proprioceptive correlations from a (realistic) virtual body and the physical body could be sufficient to elicit a FBOI with no need for additional multimodal correlations (Maselli and Slater, 2013). The current study provides more stringent evidence for the effective coupling of visual signals from the virtual body and proprioceptive signals from the real one, that is established during FBOI. It was in fact shown that proprioceptive information about the body posture can be significantly modulated by seeing a change in the posture of the virtual body, even when the physical body does not move. Furthermore, we found that if visual and proprioceptive cues about the body are not in severe conflict (i.e., when a partial spatial overlap of the virtual and real bodies is preserved) a strong FBOI can be experienced. We additionally found that in this case the FBOI has the effect of strengthening the recalibration of tactile sensations toward the virtual body, which is characteristic of OBEs. These results strongly support the earlier proposals (Moseley et al., 2012; Maselli and Slater, 2013) suggesting that a fundamental node of the complex network of brain areas involved in ownership illusions is the neural population homologous to the one hosted in area 5 of the monkey parietal cortex, that integrates visual and proprioceptive signals to encode limb position (Graziano et al., 2000).

## Summary

We have presented an experimental study conducted with the support of virtual reality, with the aim of exploring the relation between two of the important components of body self-consciousness: *ownership* and *self-location*. Our results provide direct experimental evidence that the sense of *ownership* and the sense of *self-location* can be selectively manipulated in experimentally induced full body illusions. It was shown that FBOIs and OBEs could occur independently of each other, affecting *ownership* and *self-location* respectively. It was also shown that the two illusions could co-occur affecting concurrently *ownership* and *self-location*; our results suggest that in these cases illusory changes in *ownership* and *self-location* have a synergic interaction.

We discussed how our results support the recent proposal suggesting that *self-location* is strictly related with a reshaping of the visual receptive field of proximal visuotactile neurons mapping large portions of the body's surface. On the other hand, we discussed how our results support the need for a driving activation of visuo-proprioceptive neuronal populations in full body ownership illusions.

Finally, our results moderate previous conclusions about the role of the visual perspective in ownership illusions. It was in fact shown that a FBOI could be experienced from a 3PP when the virtual body preserved a partial spatial overlap with the physical body.

### Conflict of interest statement

The authors declare that the research was conducted in the absence of any commercial or financial relationships that could be construed as a potential.

## Footnotes

^1^http://unity3d.com/

^2^http://www.nvisinc.com/index.php?id=48

^3^http://www.intersense.com/pages/20/14

^4^http://www.cs.unc.edu/Research/vrpn/index.html

^5^http://www.naturalpoint.com/optitrack/products/tracking-tools/

^6^Flat motor, model C1034-50L, by Shenzhen Linglong Electronics Co., Ltd.

## Supplementary material

The Supplementary Material for this article can be found online at:

http://www.frontiersin.org/journal/10.3389/fnhum.2014.00693/abstract

## References

[B1] Aspell J. E. Lenggenhager B. Blanke O. (2009). Keeping in touch with one's self: multisensory mechanisms of self-consciousness. PLoS ONE 4:e6488 10.1371/journal.pone.0006488 19654862 PMC2715165

[B2] Aspell J. E. Lenggenhager B. Blank O. (2012). Multisensory perception and bodily self-consciousness from out-of-body to inside-body experience, in The Neural Bases of Multisensory Processes, eds Murray M. M. Wallace M. T. (Boca Raton, FL: CRC Press), 467–481 22593890

[B3] Azañón E. Longo M. R. Soto-Faraco S. Haggard P. (2010). The posterior parietal cortex remaps touch into external space. Curr. Biol. 20, 1304–1309 10.1016/j.cub.2010.05.063 20637619

[B4] Blanke O. (2012). Multisensory brain mechanisms of bodily self-consciousness. Nat. Rev. Neurosci. 13, 556–571 10.1038/nrn3292 22805909

[B5] Blanke O. Arzy S. (2005). The out-of-body experience: disturbed self-processing at the temporo-parietal junction. Neuroscientist 11, 16–24 10.1177/1073858404270885 15632275

[B6] Blanke O. Landis T. Spinelli L. Seeck M. (2004). Out-of-body experience and autoscopy of neurological origin. Brain 127, 243–258 10.1093/brain/awh040 14662516

[B7] Blanke O. Metzinger T. (2009). Full-body illusions and minimal phenomenal selfhood. Trends Cogn. Sci. 13, 7–13 10.1016/j.tics.2008.10.003 19058991

[B8] Blanke O. Mohr C. (2005). Out-of-body experience, heautoscopy, and autoscopic hallucination of neurological origin implications for neurocognitive mechanisms of corporeal awareness and self-consciousness. Brain Res. Brain Res. Rev. 50, 184–199 10.1016/j.brainresrev.2005.05.008 16019077

[B9] Blanke O. Ortigue S. Landis T. Seeck M. (2002). Stimulating illusory own-body perceptions. Nature 419, 269–270 10.1038/419269a 12239558

[B10] Botvinick M. Cohen J. (1998). Rubber hands “feel” touch that eyes see. Nature 391, 756 10.1038/35784 9486643

[B11] Brozzoli C. Gentile G. Ehrsson H. H. (2012). That's near my hand! parietal and premotor coding of hand-centered space contributes to localization and self-attribution of the hand. J. Neurosci. 32, 14573–14582 10.1523/JNEUROSCI.2660-12.2012 23077043 PMC6621451

[B12] Bruyer R. Brysbaert M. (2011). Combining speed and accuracy in cognitive. Psychol. Belgica 51, 5–13 10.5334/pb-51-1-5

[B13] Driver J. Spence C. (1998a). Attention and the crossmodal construction of space. Trends Cogn. Sci. 2, 254–262 10.1016/S1364-6613(98)01188-7 21244924

[B14] Driver J. Spence C. (1998b). Cross-modal links in spatial attention. Philos. Trans. R. Soc. Lond. B. Biol. Sci. 353, 1319–1331 10.1098/rstb.1998.0286 9770225 PMC1692335

[B15] Duhamel J. Colby C. L. Goldberg M. E. (1998). Ventral intraparietal area of the macaque: congruent visual and somatic response properties J. Neurophysiol. 79, 126–136 9425183 10.1152/jn.1998.79.1.126

[B16] Ehrsson H. H. (2007). The experimental induction of out-of-body experiences. Science 317, 1048 10.1126/science.1142175 17717177

[B17] Ehrsson H. H. (2011). The concept of body ownership and its relation to multisensory integration, in The New Handbook of Multisensory Processes, ed Stain B. E. (Cambridge, MA: MIT Press), 775–792

[B18] Ehrsson H. H. Spence C. Passingham R. E. (2004). That's my hand! activity in premotor cortex reflects feeling of ownership of a limb. Science 305, 875–877 10.1126/science.1097011 15232072

[B19] Fogassi L. Gallese V. Fadiga L. Luppino G. Matelli M. Rizzolatti G. (1996). Coding of peripersonal space in inferior premotor cortex (area F4). J. Neurophysiol. 76, 141–157 8836215 10.1152/jn.1996.76.1.141

[B20] Graziano M. S. A. Cooke D. F. (2006). Parieto-frontal interactions, personal space, and defensive behavior. Neuropsychologia 44, 845–859 10.1016/j.neuropsychologia.2005.09.009 16277998

[B21] Graziano M. S. A. Cooke D. F. Taylor C. S. (2000). Coding the location of the arm by sight. Science 290, 1782–1786 10.1126/science.290.5497.1782 11099420

[B22] Graziano M. S. A. Gross C. G. (1993). A bimodal map of space: somatosensory receptive fields in the macaque putamen with corresponding visual receptive fields. Exp. Brain Res. 97, 96–109 8131835 10.1007/BF00228820

[B23] Grissom R. J. Kim J. J. (2012). Effect Sizes for Research: Univariate and Multivariate Applications, 2nd Edn, Vol. 44. New York, NY: Taylor and Francis Group, LLC

[B24] Guterstam A. Ehrsson H. H. (2012). Disowning one's seen real body during an out-of-body illusion. Conscious. Cogn. 21, 1037–1042 10.1016/j.concog.2012.01.018 22377139

[B25] Hohwy J. (2007). The Sense of self in the phenomenology of agency and perception. Psyche (Stuttg) 13, 1–20

[B26] Huang R.-S. Chen C. Tran A. T. Holstein K. L. Sereno M. I. (2012). Mapping multisensory parietal face and body areas in humans. Proc. Nat. Acad. Sci. U.S.A. 109, 18114–18119 10.1073/pnas.1207946109 23071340 PMC3497759

[B28] Ionta S. Heydrich L. Lenggenhager B. Mouthon M. Fornari E. Chapuis D. (2011). Multisensory mechanisms in temporo-parietal cortex support self-location and first-person perspective. Neuron 70, 363–374 10.1016/j.neuron.2011.03.009 21521620

[B28a] Iriki A. Tanaka M. Iwamura Y. (1996). Coding of modified body schema during tool use by macaque postcentralneurones. Neuroreport 7, 2325–2330 10.1097/00001756-199610020-00010 8951846

[B29] Kennett S. Eimer M. Spence C. Driver J. (2001). Tactile-visual links in exogenous spatial attention under different postures: convergent evidence from psychophysics and ERPs. J. Cogn. Neurosci. 13, 462–478 10.1162/08989290152001899 11388920

[B30] Kokkinara E. Slater M. (2014). Measuring the effects through time of the influence of visuomotor and visuotactile synchronous stimulation on a virtual body ownership illusion. Perception 43, 43–58 10.1068/p7545 24689131

[B31] Lenggenhager B. Mouthon M. Blanke O. (2009). Spatial aspects of bodily self-consciousness. Conscious. Cogn. 18, 110–117 10.1016/j.concog.2008.11.003 19109039

[B32] Lenggenhager B. Tadi T. Metzinger T. Blanke O. (2007). Video ergo sum: manipulating bodily self-consciousness. Science 317, 1096–1099 10.1126/science.1143439 17717189

[B33] Limanowski J. Blankenburg F. (2013). Minimal self-models and the free energy principle. Front. Hum. Neurosci. 7:547 10.3389/fnhum.2013.00547 24062658 PMC3770917

[B34] Limanowski J. Lutti A. Blankenburg F. (2013). The extrastriate body area is involved in illusory limb ownership. Neuroimage 86, 514–524 10.1016/j.neuroimage.2013.10.035 24185016

[B35] Lloyd D. M. Shore D. I. Spence C. Calvert G. A. (2003). Multisensory representation of limb position in human premotor cortex. Nat. Neurosci. 6, 17–18 10.1038/nn991 12483217

[B36] Longo M. R. Schüür F. Kammers M. P. M. Tsakiris M. Haggard P. (2008). What is embodiment? A psychometric approach. Cognition 107, 978–998 10.1016/j.cognition.2007.12.004 18262508

[B37] Lopez C. Halje P. Blanke O. (2008). Body ownership and embodiment: vestibular and multisensory mechanisms. Clin. Neurophysiol. 38, 149–161 10.1016/j.neucli.2007.12.006 18539248

[B38] Makin T. R. Holmes N. P. Ehrsson H. H. (2008). On the other hand: dummy hands and peripersonal space. Behav. Brain Res. 191, 1–10 10.1016/j.bbr.2008.02.041 18423906

[B39] Maravita A. Iriki A. (2004). Tools for the body (schema). Trends Cogn. Sci. 8, 79–86 10.1016/j.tics.2003.12.008 15588812

[B40] Maravita A. Spence C. Driver J. (2003). Multisensory integration and the body schema: close to hand and within reach. Curr. Biol. 13, R531–R539 10.1016/S0960-9822(03)00449-4 12842033

[B41] Maravita A. Spence C. Kennett S. Driver J. (2002). Tool-use changes multimodal spatial interactions between vision and touch in normal humans. Cognition 83, B25–B34 10.1016/S0010-0277(02)00003-3 11869727

[B42] Maselli A. Slater M. (2013). The building blocks of the full body ownership illusion. Front. Hum. Neurosci 7:83 10.3389/fnhum.2013.00083 23519597 PMC3604638

[B43] Moseley G. L. Gallace A. Spence C. (2012). Bodily illusions in health and disease: physiological and clinical perspectives and the concept of a cortical “body matrix.” Neurosci. Biobehav. Rev. 36, 34–46 10.1016/j.neubiorev.2011.03.013 21477616

[B44] Moseley G. L. Olthof N. Venema A. Don S. Wijers M. Gallace A. (2008). Psychologically induced cooling of a specific body part caused by the illusory ownership of an artificial counterpart. Proc. Nat. Acad. Sci. U.S.A. 105, 13169–13173 10.1073/pnas.0803768105 18725630 PMC2529116

[B45] Pavani F. Spence C. Driver J. (2000). Visual capture of touch: out-of-the-body experiences with rubber gloves. Psychol. Sci. 11, 353–359 10.1111/1467-9280.00270 11228904

[B46] Peck T. C. Seinfeld S. Aglioti S. M. Slater M. (2013). Putting yourself in the skin of a black avatar reduces implicit racial bias. Conscious. Cogn. 22, 779–787 10.1016/j.concog.2013.04.016 23727712

[B47] Perneger T. V. (1998). What's wrong with Bonferroni adjustments. BMJ 316, 1236–1238 9553006 10.1136/bmj.316.7139.1236 PMC1112991

[B48] Petkova V. I. Björnsdotter M. Gentile G. Jonsson T. Li T.-Q. Ehrsson H. H. (2011a). From part- to whole-body ownership in the multisensory brain. Curr. Biol. 21, 1118–1122 10.1016/j.cub.2011.05.022 21683596

[B49] Petkova V. I. Ehrsson H. H. (2008). If I were you: perceptual illusion of body swapping. PLoS ONE 3:e3832 10.1371/journal.pone.0003832 19050755 PMC2585011

[B50] Petkova V. I. Khoshnevis M. Ehrsson H. H. (2011b). The perspective matters! Multisensory integration in ego-centric reference frames determines full-body ownership. Front. Psychol. 2:35 10.3389/fpsyg.2011.00035 21687436 PMC3108400

[B51] Pomés A. Slater M. (2013). Drift and ownership toward a distant virtual body. Front. Hum. Neurosci. 7:908 10.3389/fnhum.2013.00908 24399960 PMC3872309

[B52] Salomon R. Lim M. Pfeiffer C. Gassert R. Blanke O. (2013). Full body illusion is associated with widespread skin temperature reduction. Front. Behav. Neurosci. 7:65 10.3389/fnbeh.2013.00065 23898244 PMC3724056

[B53] Salomon R. van Elk M. Aspell J. E. Blanke O. (2012). I feel who I see: visual body identity affects visual-tactile integration in peripersonal space. Conscious. Cogn. 21, 1355–1364 10.1016/j.concog.2012.06.012 22832215

[B54] Sereno M. I. Huang R.-S. (2014). Multisensory maps in parietal cortex. Curr. Opin. Neurobiol. 24, 39–46 10.1016/j.conb.2013.08.014 24492077 PMC3969294

[B55] Serino A. Alsmith A. Costantini M. Mandrigin A. Tajadura-jimenez A. Lopez C. (2013). Bodily ownership and self-location: components of bodily. Conscious. Cogn. 22, 1239–1252 10.1016/j.concog.2013.08.013 24025475

[B57] Serino A. Pizzoferrato F. Làdavas E. (2008). Viewing a face (especially one's own face) being touched enhances tactile perception on the face. Psychol. Sci. 19, 434–438 10.1111/j.1467-9280.2008.02105.x 18466402

[B58] Shapiro S. S. Wilk M. B. (1965). An analysis of variance test for normality (complete samples). Biometrica 52, 591–611

[B59] Shokur S. O'Doherty J. E. Winans J. A. Bleuler H. Lebedev M. A. Nicolelis M. A. L. (2013). Expanding the primate body schema in sensorimotor cortex by virtual touches of an avatar. Proc. Nat. Acad. Sci. U.S.A. 110, 15121–15126 10.1073/pnas.1308459110 23980141 PMC3773736

[B60] Shore D. I. Barnes M. E. Spence C. (2006). Temporal aspects of the visuotactile congruency effect. Neurosci. Lett. 392, 96–100 10.1016/j.neulet.2005.09.001 16213655

[B61] Slater M. Spanlang B. Sanchez-Vives M. V. Blanke O. (2010). First person experience of body transfer in virtual reality. PLoS ONE 5:e10564 10.1371/journal.pone.0010564 20485681 PMC2868878

[B62] Spence C. Kingstone A. Shore D. I. Gazzaniga M. S. (2001). Representation of Visuotactile space in the split brain. Psychol. Sci. 12, 90–93 10.1111/1467-9280.00316 11294236

[B63] Spence C. Pavani F. Driver J. (2004). Spatial constraints on visual—tactile cross-modal distractor congruency effects. Cogn. Affect. Behav. Neurosci 4, 148–169 10.3758/CABN.4.2.148 15460922

[B64] Spence C. Pavani F. Maravita A. Holmes N. P. (2008). Multi-sensory interactions, in Haptic Rendering: Foundations, Algorithms, and Applications, eds Lin M. C. Otaduy M. A. (Wellesley, MA: AK Peters), 21–52

[B65] Townsend J. T. Ashby F. G. (1983). The stochastic Modeling of Elementary Psychological Processes. *The American Journal of Psychology*, Vol. 98. Cambridge: Cambridge University Press

[B66] Tsakiris M. Hesse M. D. Boy C. Haggard P. Fink G. R. (2007). Neural signatures of body ownership: a sensory network for bodily self-consciousness. Cereb. Cortex 17, 2235–2244 10.1093/cercor/bhl131 17138596

[B67] Tsakiris M. Longo M. R. Haggard P. (2010). Having a body versus moving your body: neural signatures of agency and. Neuropsychologia 48, 2740–2749 10.1016/j.neuropsychologia.2010.05.021 20510255

[B68] Tukey J. Ws. (1977). Exploratory Data Analysis. Reading, MA: Addison-Wesley

[B69] Zopf R. Savage G. Williams M. A. (2010). Crossmodal congruency measures of lateral distance effects on the rubber hand illusion. Neuropsychologia 48, 713–725 10.1016/j.neuropsychologia.2009.10.028 19913040

[B70] Zopf R. Truong S. Finkbeiner M. Friedman J. Williams M. A. (2011). Viewing and feeling touch modulates hand position for reaching. Neuropsychologia 49, 1287–1293 10.1016/j.neuropsychologia.2011.02.012 21320514 PMC3086579

